# Outcomes of Nigeria's HIV/AIDS Treatment Program for Patients Initiated on Antiretroviral Treatment between 2004-2012

**DOI:** 10.1371/journal.pone.0165528

**Published:** 2016-11-09

**Authors:** Ibrahim Dalhatu, Dennis Onotu, Solomon Odafe, Oseni Abiri, Henry Debem, Simon Agolory, Ray W. Shiraishi, Andrew F. Auld, Mahesh Swaminathan, Kainne Dokubo, Evelyn Ngige, Chukwuemeka Asadu, Emmanuel Abatta, Tedd V. Ellerbrock

**Affiliations:** 1 Division of Global HIV/AIDS, Center for Global Health, U.S. Centers for Disease Control & Prevention, Abuja, Nigeria; 2 School of Biomedical Informatics, University of Texas, Houston, Texas, United States of America; 3 Division of Global HIV/AIDS, Center for Global Health, U.S. Centers for Disease Control & Prevention, Atlanta, Georgia, United States of America; 4 National AIDS & STIs Control Program, Federal Ministry of Health, Abuja, Nigeria; Tulane University School of Public Health and Tropical Medicine, UNITED STATES

## Abstract

**Background:**

The Nigerian Antiretroviral therapy (ART) program started in 2004 and now ranks among the largest in Africa. However, nationally representative data on outcomes have not been reported.

**Methods:**

We evaluated retrospective cohort data from a nationally representative sample of adults aged ≥15 years who initiated ART during 2004 to 2012. Data were abstracted from 3,496 patient records at 35 sites selected using probability-proportional-to-size (PPS) sampling. Analyses were weighted and controlled for the complex survey design. The main outcome measures were mortality, loss to follow-up (LTFU), and retention (the proportion alive and on ART). Potential predictors of attrition were assessed using competing risk regression models.

**Results:**

At ART initiation, 66.4 percent (%) were females, median age was 33 years, median weight 56 kg, median CD4 count 161 cells/mm^3^, and 47.1% had stage III/IV disease. The percentage of patients retained at 12, 24, 36 and 48 months was 81.2%, 74.4%, 67.2%, and 61.7%, respectively. Over 10,088 person-years of ART, mortality, LTFU, and overall attrition (mortality, LTFU, and treatment stop) rates were 1.1 (95% confidence interval (CI): 0.7–1.8), 12.3 (95%CI: 8.9–17.0), and 13.9 (95% CI: 10.4–18.5) per 100 person-years (py) respectively. Highest attrition rates of 55.4/100py were witnessed in the first 3 months on ART. Predictors of LTFU included: lower-than-secondary level education (reference: Tertiary), care in North-East and South-South regions (reference: North-Central), presence of moderate/severe anemia, symptomatic functional status, and baseline weight <45kg. Predictor of mortality was WHO stage higher than stage I. Male sex, severe anemia, and care in a small clinic were associated with both mortality and LTFU.

**Conclusion:**

Moderate/Advanced HIV disease was predictive of attrition; earlier ART initiation could improve program outcomes. Retention interventions targeting men and those with lower levels of education are needed. Further research to understand geographic and clinic size variations with outcome is warranted.

## Introduction

Studies from a wide range of resource settings have reported a consistent pattern of positive benefits from antiretroviral therapy (ART), including reduced risk of HIV transmission, reduced morbidity, and mortality, and improved overall health and quality of life in patients adhering to treatment and keeping appointments[1–7].

Nigeria, with a population of approximately 177 million people and about 250 different ethnic groups [8] is home to about one in five people living on the African continent, making it Africa’s most populous nation. Since the first AIDS patient was diagnosed in 1985 [9], national adult HIV prevalence has risen from about 1.8% in 1991 to about 4.1% in 2010[10] and was an estimated 3.2% in 2014[11]. Currently, about 3.4 million people are estimated to be living with HIV/AIDS [12], about 1.5 million of whom are in need of ART[13].

The Government of Nigeria, with support from the U.S. President’s Emergency Plan for AIDS Relief (PEPFAR), and other international and domestic donors, has dramatically scaled up ART services in Nigeria. The period between 2006 and 2010 corresponds to a time of major ART program expansion in Nigeria; the number of patients initiated on ART increased from 90,008 in 2006 [14] to an estimated 300,000 in 2009 [15]. At the end of 2012, a total of 491, 021 (29%) of the 1.5 million people in need of ART were receiving therapy [16]. Collaboration with stakeholders, decentralization, and integration with primary care systems have been important strategies deployed by implementing partners to rapidly improve access to HIV care and treatment in the country.

Several studies to demonstrate HIV/AIDS treatment outcomes have been conducted in Nigeria [17–19]; however, these studies did not provide nationally representative data. We conducted a multicenter study using nationally representative data to assess ART outcomes and its determinants.

## Materials and Methods

### Study Design and Setting

The study was a retrospective cohort analysis of routinely collected data from PEPFAR supported ART facilities that had initiated more than 50 patients on ART by December 31, 2010. All adults, regardless of outcome at the time of chart abstraction, aged 15 years or older at the time of enrollment on ART at one of the eligible sites, on or after January 1, 2004, and at least 12 months prior to the date of chart abstraction, were eligible for inclusion in the evaluation.

### Antiretroviral Treatment Outcomes and definitions

In this study, treatment stop refers to a physician recommended temporary stop of ART for any reason; usually, such treatment interruptions are due to severe adverse drug reaction such as immune reconstitution syndrome (IRIS) [20], and ART is usually ‘restarted’ as soon as tolerability has been established. ‘Treatment stop’ was considered an outcome in this study irrespective of ART restart status. Stopping ART has been reported to result in viral rebound, immune decompensation, and progression of disease leading to worsening of morbidity and increased the risk of death [21–23]. Therefore, even planned interruptions of ART are not generally recommended[24]. LTFU refers to failure to return for drug refill appointment for at least 90 days from the last given appointment date and attempts at tracking failed; while mortality refers to known deaths. We defined attrition as treatment stop, LTFU, and mortality [25]. Retention was defined as being alive and still on ART at the time of last appointment [25]. Patients’ Hemoglobin concentration results at baseline and follow-up were used in classification for anemia based on WHO recommendations[26]. In males, hemoglobin concentration levels of ≥13, 11–12.9, 8–10.9 and <8g/dl were considered not anemic, mild, moderate, or severely anemic respectively; in females, hemoglobin concentration levels of ≥12, 11–11.9, 8–10.9and <8g/dl were considered not anemic, mild, moderate, or severely anemic respectively[26].

### Sample Size Determination

The distribution of people living with HIV in Nigeria vary markedly due to the wide disparity in HIV prevalence rates by state and geographical region; estimates range from 1.0% in the lowest burdened state to 12.7% in the highest burdened State[27]. Subsequently, the number of patients receiving treatment in each site vary and range from 52 to 11,447 per site. To minimize the loss of effectiveness due to anticipated intra-stratum correlation, we assumed a design effect of 3.0, so that a 95% CI equal to the sample proportion +2.5% is produced. A review of retention rates by Rosen et al reported average retention rates of HIV-1-infected patients in African ART programs to be 79.1%, 75.0% and 61.6% at 6, 12, and 24 months respectively[25]. We calculated the minimum sample size using an assumption that about 75% of patients will be retained on treatment 12 months after ART start [25]. We estimated that a minimum sample size of approximately 3,500 medical records from ART sites will be required to determine the retention rates at 12 months after ART initiation.

### Site and Sample Selection

Based on a calculated sample size of 3,500 patient charts; 100 charts per selected ART site. All PEPFAR supported ART sites in Nigeria that have initiated more than 50 patients on ART by Dec 31, 2010, were included in the evaluation. Of these, 35 ART facilities were randomly selected using probability proportional to size sampling (PPS), where size was based on the number of patients initiated on ART by Dec 31, 2010. Sites were implicitly stratified based on their location in the six geopolitical zones of Nigeria (North Central, North East, North West, South East, South-South, and South West) and size (small [<500], medium [500–1,499] and large [≥1,500]). We designed a program using SAS 9.1, to randomly sample the required number of sites from each sub-stratum, so that the probability of selection was proportional to the size of each site without replacement. A total of 35 out of 322 ART sites were selected. In each site, the study team developed sampling frames consisting of patient identification numbers (IDs) of all patients initiated on ART from January 2004 to December 2012. We used these sampling frames to select 100 patient identification numbers by simple random sampling. The charts selected were subsequently abstracted for each site.

Once at a site, the abstractors pulled the charts and screened them for eligibility, recording on a screening log if a chart was: 1) missing, 2) ineligible, or 3) eligible and abstracted. Replacement numbers were generated for those charts that were either ineligible or were missing. Initiation on ART was determined by reviewing ART registers, patient charts, and confirming that a patient is/was on antiretrovirals (ARVs).

### Data Collection and Management

Data abstractors pulled the charts and screened them using the study screening log and the missing chart log. If a chart was eligible, the data abstractor would extract the data using the ART data abstraction tools specially designed for the study. The abstractors pulled data from the clinical record and, when applicable, from the laboratory and pharmacy logs. On-site data verification was conducted before the data abstraction tools were sent, with the screening logs, to the data coordination center, where a data-management team double entered and verified all data entry using CSPro (Census and Survey Processing System).

### Antiretroviral Treatment Guideline and Program

The treatment guidelines in Nigeria are based on World Health Organization (WHO) recommendations [28, 29]. Hence, eligibility criteria and the preferred first-line ART regimens changed within the study period due to changes in national guidelines. Between 2003 and 2010, HIV-positive patients assessed as having WHO stage 4, stage 3 with CD4 ≤350 and those with a CD4 count <200 cells/mm3 (irrespective of WHO staging) were considered eligible for ART[28, 30]. After 2010, all patients with CD4 ≤350 cells/mm3 (irrespective of WHO staging) and those in WHO Clinical stage 3 or 4, were eligible for ART[31].

First-line ART is the initial regimen prescribed for an ART-naïve patient, while second-line ART is the next regimen used after treatment failure or patient intolerance of first-line ART. In general, first line regimen included two nucleoside reverse transcriptase inhibitors (NRTI) and one non-nucleoside reverse transcriptase inhibitor, while second-line regimens contained a protease inhibitor and two NRTIs. In 2003–2006, the preferred first-line regimen was Stavudine (D4T) or Zidovudine (AZT), Lamivudine (3TC) and Nevirapine (NVP)[32]. Between 2007–2010, the preferred first-line regimen was Zidovudine (AZT), Lamivudine (3TC) and Nevirapine (NVP) (with Zidovudine (AZT), Lamivudine (3TC) and Efavirenz(EFV) as an alternative)[31]; and after 2010, the preferred first-line regimen was Tenofovir (TDF), Lamivudine (3TC) or Emtricitabine (FTC), and Efavirenz(EFV)[31]. These changes were aimed at addressing safety profile concerns, issues of pill burden, the efficacy of regimen combination, as well as compliance with international standards.

### Laboratory monitoring during follow-up

Plasma viral load (VL) monitoring is the gold standard for monitoring response to ART and also a tool to reinforce adherence [33, 34]. However, challenges with access to VL testing limits its use in resource-limited settings[34]. The WHO 2013 consolidated guidelines have recommended use of CD4 cell count measurement and clinical outcomes for monitoring ART in the absence of viral load[35]. During the study period, monitoring of CD4 cell count was the mainstay for laboratory monitoring in Nigeria[36, 37]. In Nigeria, CD4 cell count in healthy individuals ranges between 365/mm^3^ to 1571/mm^3^ of blood [38]. Patients on ART were expected to receive 6 monthly CD4 count tests to evaluate progress on ART [36, 37]. All ART sites in this study had access to CD4 assays.

### Data analysis

Data analyses were performed using Stata 13[39]. We controlled for the complex survey design (i.e., clustering and weighting) using svyset and svy procedures in Stata. Multiple imputation by chained equations[40, 41], was used to impute missing baseline sociodemographic and clinical data. We assumed that missing data were missing at random (MAR). All patients had complete time-to-event data. After an initial 5 imputation to test convergence, we increased imputation to 60 and burn-in iteration by 50. We carried out imputation sensitivity analysis and checked the fit of the imputation model using the Stata command “Midiagplots”[42]. For all analyses using imputed data, estimates were combined across the imputed datasets based to Rubin's rules[43]. The main outcome measures were mortality and LTFU. The predictor variables analyzed were patients’ sociodemographic and clinical characteristics at ART initiation. Potential predictors associated with time to mortality and time to LTFU were assessed using competing risks regression models described by Fine and Gray [44]. Competing risk regression models was used to estimate crude and adjusted hazard ratios (AHR) and 95% CI for covariates of interest. Competing risk for death was LTFU and treatment stop. Similarly, competing risk for LTFU was mortality and treatment stop. Competing risk models were weighted and clustering was accounted for using a cluster adjusted sandwich variance estimator[45]. We adjusted for the effects of selected baseline and clinical characteristics in our multivariable regression models. The variables included in the regression model for LTFU and mortality were those previously reported to be associated with either, attrition, LTFU, or mortality in previous studies [18, 46–51].

The life table for survival function in Stata[52] was used to estimate retention rates. We used the ‘stptime’ function in Stata[53] to estimate person-time and incidence rates per 100py for treatment stop, mortality, LTFU, and attrition by duration on ART. In the cumulative failure table, individuals that have been on ART for between zero and 3 months contributed to the person-time for 0 to 0.3. While those that have been on ART for greater than 3 months but less than or equal to 6 months contributed to the time 0.3 to 0.5 years and so on. Individuals who were on ART for less than 3 months will not contribute to the person-time 0.3 to 0.5 years. Therefore, patients that died, or LTFU in the preceding time frame and those that have been on ART for less than the minimum time specified will not contribute to the subsequent analysis time. A patient may contribute to multiple rows in the analysis if they have been on treatment long enough and retained on ART. Kaplan-Meier curves were used to examine the cumulative probability of retention over time stratified by baseline variables. The differences in survival distributions by subgroups of baseline variables were tested using the stcox function in Stata. During bivariate analyses, we used Rao-Scott chi-square test after accounting for the study design to examine associations between categorical variables.

The analyses comparing baseline, and follow-up weights, and CD4 cell counts, and differences by gender were performed using linear regression after accounting for the survey design, and Wald post-estimation test[54]. The incidence rates of, mortality, LTFU, and overall attrition were expressed as the number of patients with at least one occurrence of the given event per 100 person-years (py). We calculated incidence rates per 100 py and respective 95% CI by patients’ sociodemographic and clinical characteristics and compared rates within subgroups of variables using Poisson regression, after rescaling the exposure variable so that the incidence rate (IR) would be per 100py.

### Ethics statement

The study protocol was reviewed and approved by National Health Research Ethics Committee of Nigeria (NHREC) and the US Centers for Disease Control and Prevention (CDC) Atlanta Institutional Review Board (IRB). Patient informed consent was not required as only routine, anonymous, operational monitoring data were collected and analyzed.

## Results

### Patients’ sociodemographic characteristics at ART start

Data from eligible medical records of 3,496 adult ART patients were abstracted and analyzed. Table 1 summarizes analysis results for both original and imputed datasets for sociodemographic and clinical characteristics of patients. In the following sections, weighted imputed data are reported unless otherwise stated. Of the 3,496 patient records abstracted, 66.4% were females. The median age at ART initiation was 33 (interquartile range [IQR]: 28–40) years, but higher among males 37 (IQR: 32–44) compared with females 30 (IQR: 25–37). The majority of the study population was married (62.2%) and employed (61.1%). With respect to patient educational status, 25.2% and 40.3% had primary and secondary education, respectively. A substantial percentage (56.4%) of the patients was initiated on ART between 2007 and 2009.

**Table 1:** Patients Sociodemographic and Clinical Characteristics.

	Original Data			Following Multiple Imputation (N = 3,496)
Patient characteristics	Un-weighted Frequency of Observation	Un-weighted Total	Weighted Percentages with (95% CI) OR Median with (IQR)	Weighted Median with IQR*, OR Percentages with (95% CI)
Sex^Ϯ^ No., N, %, (95% CI)				
Females	2,320	3,496	66.4% (63.8–68.9)	66.4% (63.8–68.9)
Males	1,176	3,496	33.4% (31.2–36.2)	33.4% (31.2–36.2)
Age, No., N, %, (95% CI)				
15–24 years	391	3,494	11.2% (9.6–13.0)	10.6% (9.0–12.2)
25–34 years	1,492	3,494	42.7% (40.7–44.8)	42.4% (40.1–44.6)
35–44 years	1,053	3,494	30.1% (28.4–31.9)	30.7% (28.8–32.6)
45–54 years	424	3,494	12.1% (10.8–13.6)	12.4% (11.0–13.7)
>55 years	134	3,494	3.8% (3.2–4.6)	3.9% (3.2–4.6)
observations missing data**	2	3,496	0.1%	
Median Age (years) No., N, median (IQR)				
Both sexes	3,494	3,494	34 (28–40)	33 (28–40)
Females	2,320	2,320	31 (26–38)	30 (25–37)
Males	1,176	1,176	38 (32–44)	37 (32–44)
Marital No., N, %, (95% CI)				
Single	649	3,410	19.0% (16.2–22.2)	20.6% (17.2–24.0)
Married	2,172	3,410	63.7% (60.9–66.4)	62.2% (59.2–65.2)
Divorced/separated	219	3,410	6.4% (5.3–7.9)	6.5% (5.2–7.9)
Widowed	370	3,410	10.9% (9.4–12.4)	10.6% (9.3–11.9)
observations missing data**	86	3,496	2.5%	
Employment status No., N, %, (95% CI)				
Employed	1,797	3,076	58.4% (50.5–65.9)	61.1% (53.4–68.8)
Not currently employed	1,279	3,076	41.6% (34.1–49.5)	38.9% (31.2–46.6)
observations missing data**	420	3,496	12.0%	
Educational Status No., N, %, (95% CI)				
Tertiary	639	3,009	21.2% (17.9–25.0)	22.7% (19.1–26.2)
Secondary	1,173	3,009	39.0% (34.9–43.3)	40.3% (36.1–44.4)
Primary	842	3,009	28.0% (23.3–33.2)	25.2% (21.5–28.9)
No education	355	3,009	11.8% (7.7–17.6)	11.9% (6.3–17.4)
observations missing data**	487	3,496	13.9%	
Clinic size^Ϯ^ No., N, %, (95% CI)				
Large (≥1,500)	2,296	3,496	65.7% (47.9–80.0)	70.9% (54.5–87.2)
Medium (500–1499)	800	3,496	22.9% (11.5–40.5)	18.8% (5.2–32.2)
Small (<500 patients)	400	3,496	11.4% (4.1–27.9)	10.4% (0.8–21.6)
Region^Ϯ^				
North Central	1,200	3,496	34.3% (20.1–52.1)	34.3% (20.1–52.1)
North East	399	3,496	11.4% (4.1–27.8)	11.4% (4.1–27.8)
North West	500	3,496	14.3% (5.8–31.1)	14.3% (5.8–31.1)
South East	399	3,496	11.4% (4.1–27.8)	11.4% (4.1–27.8)
South-South	499	3,496	14.3% (5.8–31.1)	14.3% (5.8–31.1)
South West	499	3,496	14.3% (5.8–31.1)	14.3% (5.8–31.1)
Baseline Cd4 count, No., N, %, (95% CI)				
>350 cells/mm^3^	201	2,876	7.0% (5.5–8.9)	7.2% (5.3–9.1)
201–350 cells/mm^3^	872	2,876	30.3% (27.6–33.2)	29.6% (26.6–32.6)
101–200 cells/mm^3^	907	2,876	31.5% (29.3–33.9)	32.2% (29.9–34.5)
< = 100 cells/mm^3^	896	2,876	31.2% (28.2–34.3)	31.0% (27.6–34.4)
observations missing data**	620	3,496	17.7%	
Baseline Median CD4 Count No., N, median (IQR)				
Both sexes	2,876	2,876	159 (81–248)	161 (83–254)
Females	1,891	1,891	166 (88–260)	170 (92.7–265.4)
Males	985	985	141 (68–223)	143 (69.9–229.5)
WHO Clinical Stage No., N, %, (95% CI)				
Stage I	845	3,264	25.9% (21.1–31.3)	26.0% (20.5–31.4)
Stage II	850	3,264	26.0% (22.6–29.8)	27.0% (23.1–31.0)
Stage III	1,323	3,264	40.5 (34.6–46.7)	39.0% (32.5–45.4)
Stage IV	246	3,264	7.5% (5.1–11.0)	8.1% (4.9–11.2)
observations missing data**	232	3,496	6.6%	
				
Baseline functional status				
Asymptomatic	2,151	3,160	68.07% (59.6–75.5)	62.5% (52.9–72.1)
Symptomatic	909	3,160	28.8% (22.0–36.7)	33.1 (24.7–41.6)
Bedridden	100	3,160	3.2% (2.1–4.8)	4.3% (2.2–6.4)
observations missing data**	336	3496	9.6%	
NRTI Backbone				
D4T	739	3,242	22.8% (16.6–30.5)	23.1% (15.0–31.2)
TDF	746	3,242	23.0% (17.5–29.6)	23.6% (16.9–30.2)
AZT	1,703	3,242	52.5% (44.6–60.3)	51.1% (43.4–58.9)
Sub-optimal	54	3,242	1.7% (0.9–2.9)	2.1% (1.0–3.4)
observations missing data**	254	3,496	7.3%	
Baseline hemoglobin category No., N, %, (95% CI)				
Not Anemic	372	1,700	21.9% (18–26.3)	21.4% (16.8–26)
Mild Anemia	686	1,700	40.4% (37.7–43.0)	19% (16.8–21.3)
Moderate anemia	437	1,700	25.7% (22.7–29.0)	44.1% (40.4–47.9)
Severe anemia	205	1,700	12.1% (9.6–15.0)	15.5% (11.2–19.6)
observations missing data**	1796	3,496	51.5%	
Baseline Median hemoglobin No., N, median (IQR)				
Both sexes	1,700	1,700	10.3 (9–12)	10.5 (8.97–12.09)
Females	1,096	1,700	10.0 (8.9–11.3)	10.19 (8.76–11.7)
Males	604	1,700	11.0 (9.3–13.0)	11.26 (9.56–13.03)
Baseline weight category No., N, %, (95% CI)				
60 kg	1,155	3,286	35.2% (31.2–39.3)	36.4% (31.8–41)
45–60 kg	1,721	3,286	52.4% (49.4–55.5)	50.7% (47.5–54)
<45kg	410	3,286	12.5% (10.5–14.8)	12.9% (10.2–15.5)
observations missing data**	210	3,496	6.0%	
Baseline Median weight No., N, median (IQR)				
Both sexes	3,286	3,286	56 (49–65)	56 (49–65)
Females	2,179	2,179	54 (47–62)	54 (47–62)
Males	1,107	1,107	60 (54–68)	60 (54–68)
CTX at ART initiation				
Yes	2,766	3,365	82.2% (74.9–87.7)	80.1% (72.3–87.8)
No	599	3,365	17.8% (12.3–25.1)	19.9% (12.2–27.7)
observations missing data**	131	3,496	3.7%	
Year of ART start ^Ϯ^ No., N, %, (95% CI)				
2004–2006	390	3,496	11.2% (7.1–12.0)	11.2 (7.1–12.0)
2007–2009	1,970	3,496	56.4% (51.6–61.0)	56.4 (51.6–61.0)
2010–2012	1,136	3,496	32.5% (27.5–38.0)	32.5 (27.5–38.0)

Abbreviations: CI, confidence interval; IQR, interquartile range; WHO, World Health Organization; Kgs, kilograms; ART, antiretroviral therapy; D4T, stavudine; 3TC, lamivudine; NVP, nevirapine; EFV, efavirenz; AZT, zidovudine; ABC, abacavir; CTX, co-trimoxazole.

**Unweighted sample estimate.

*Median and IQR calculated across 60 imputed datasets.

ϮVariables with complete data.

### Baseline clinical characteristics at start of ART

At ART initiation, median CD4 cell count was 161 (IQR: 83–254) cells/mm^3^, with the majority of patients (63.1%) having a CD4 cell count ≤200 cells/mm^3^. The median CD4 cell count for females of 170 (IQR: 92.7–265.4) cells/mm^3^ was higher than that for males (143; IQR: 69.9–229.5) cells/mm^3^, p<0.01. The median weight for all population was 56kg (IQR: 49–65), about 12.9% of the study population had baseline weight <45kg. More than a third of patients (39.0%) were initiated on ART at WHO stage III while only 8.1% were initiated at stage IV. At ART start, 62.5% of study patients had asymptomatic functional status, while 4.3% were bedridden. The median hemoglobin at ART initiation was 11.3 (IQR: 9.6–13.0) g/dL and 10.2 (IQR: 8.8–11.7) g/dL in males and females, respectively. A substantial proportion of the patients were moderately anemic (44.1%), while 15.5% had severe anemia.

### Mortality and LTFU rates during follow-up

Table 2 illustrates attrition and retention rates over the study period. Over 10,088 py on ART, mortality, LTFU and overall attrition (mortality, LTFU, and treatment stop) rates were 1.1 (95% CI: 0.7–1.8), 12.3 (95% CI: 8.9–17.0), and 13.9 (95%CI: 10.4–18.5) per 100 (py) respectively. Highest mortality and LTFU rates of 6.5/100py, and 47.5/100py, respectively were witnessed in the first 3 months on ART.

**Table 2:** Treatment stop, LTFU, Mortality, attrition and retention rates by time on ART.

Time On ART (Yrs.)	Patients’ beginning total	person-years	Treatment stop events	Treatment stop Rate/100py	LTFU events	LTFU Rate/100py	Mortality events	Mortality Rate/100py	Attrition events	Attrition Rate/100py	Retention rates (%)
**0–.3**	3496	791	11	1.4	376	47.5	51	6.5	439	55.4	88.5
**.3–.5**	3084	745	6	0.8	103	13.9	10	1.4	119	16.0	85.3
**.5–1**	2963	1414	10	0.7	122	8.6	12	0.8	144	10.2	81.2
**1–2**	2790	2479	10	0.4	188	7.6	14	0.6	211	8.5	74.4
**2–3**	2231	1909	6	0.3	187	9.8	15	0.8	207	10.8	67.2
**3–4**	1651	1352	3	0.2	116	8.6	3	0.2	122	9.0	61.7
**4–5**	1058	808	1	0.1	81	10	2	0.3	84	10.4	55.9
**5–6**	526	374	2	0.5	31	8.3	3	0.8	36	9.7	51.2
**6–7**	205	170	1	0.6	31	18.6	0	0	33	19.2	42.6
**> 7**	77	47	0	0	8	16.9	0	0	8	16.9	36.4
**total**		10088	50	0.5	1243	12.3	110	1.1	1403	13.9	

### Mortality and LTFU rates changes by patients’ characteristics

Tables 3 and 4 illustrate LTFU and mortality rates per 100py by patients’ sociodemographic and clinical characteristics respectively. The LTFU rates/100py show variation by patients’ characteristics. The rates were higher in males, patients with less than tertiary level education, as well as in patients on suboptimal regimen. Similarly, the LTFU rates were higher in patients receiving treatment in small clinics, and those in the South-South, and Northeast regions, as well as among patients initiated on ART between 2010 and 2012. Additionally, the LTFU rates appear to increase with increasing disease severity. The rates increased with reducing baseline CD4 cell count, baseline weights, and baseline hemoglobin concentration. Furthermore, we observed higher mortality rates/100py among males, as well as in patients with signs of severe immunosuppression evidence by baseline CD4 cell count <100 cells/mm^3^, stage III and IV disease, severe anemia and baseline weights less than 45kg as well as in patients with symptomatic or bedridden functional status.

**Table 3:** Patient characteristics at antiretroviral therapy initiation associated with LTFU.

		Lost to follow up				
		Following multiple imputation (N = 3,496)				
Patient characteristics	Rate/100py (95% CI)	P values	Un-adjusted Haz. Ratio (95%CI)	P values	^#^Adjusted Haz. Ratio (95%CI)	P values
**Sex**						
**Female**	11.68 (10.7–12.7)		1.0		1.0	
**Male**	13.56 (12.1–15.1)	<0.01	1.13 (0.95–1.34)	0.16	1.36 (1.16–1.59)	<0.01
**Employment Status**						
**Employ**	10.96 (10.0–12.0)		1.0		1.0	
**not currently employed**	14.66 (13.3–16.1)	0.58	1.31 (0.96–1.77)	0.08	1.04 (0.84–1.30)	0.72
**Educational status**						
**Tertiary**	9.45 (8.18–11.0)		1.0		1.0	
**Secondary school**	11.1 (10.0–12.4)	0.04	1.09 (0.90–1.32)	0.35	1.15 (0.96–1.4)	0.13
**Primary school**	14.4 (12.7–16.4)	<0.01	1.39 (1.12–1.7)	0.00	1.41 (1.14–1.7)	<0.01
**None**	19.7 (16.8–23.0)	<0.01	1.87 (1.33–2.64)	0.00	1.59 (1.14–2.2)	0.01
**CD4 at ART Start**						
**>350**	9.4 (7.4–12.1)		1.0		1.0	
**201–350**	10.6 (9.4–11.8)	0.10	0.95 (0.65–1.4)	0.77	0.98 (0.60–1.27)	0.92
**101–200**	11.6(10.4–12.9)	0.01	1.18 (0.78–1.8)	0.43	1.19 (0.89–1.59)	0.24
**<100**	13.8(12.4–15.3)	0.01	1.16 (0.74–1.8)	0.51	1.1 (0.79–1.47)	0.64
**WHO Clinical stage at ART start**						
**Stage I**	7.9 (6.9–9.0)		1.0		1.0	
**Stage II**	10.0 (8.9–11.3)	0.90	1.23 (0.93–1.7)	0.14	0.91 (0.74–1.12)	0.38
**Stage III**	14.2 (13.1–15.5)	0.84	1.59 (1.22–2.07)	0.00	0.96 (0.76–1.22)	0.76
**Stage IV**	20.5 (17.2–25.4)	0.95	2.10 (1.22–3.63)	0.00	1.04 (0.77–1.40)	0.80
**Baseline weight**						
**>60 kg**	8.6 (7.7–9.6)		1.0		1.0	
**45–60 kg**	12.5 (11.5–13.5)	0.03	1.34 (1.16–1.56)	0.00	1.16 (0.97–1.4)	0.11
**<45 kg**	19.9 (17.3–23.1)	<0.01	2.07 (1.59–2.72)	0.00	1.65 (1.23–2.21)	0.00
**Baseline hemoglobin**						
**Not anemic**	8.8 (7.5–10.5)		1.0		1.0	
**Mild anemia**	10.9 (9.1–13.0)	0.01	1.30 (0.98–1.71)	0.06	1.21 (0.93–1.59)	0.16
**Moderate anemia**	13.0 (11.8–14.4)	<0.01	1.60 (1.26–2.05)	0.00	1.46 (1.13–1.90)	0.01
**Severe anemia**	14.5 (12.0–17.4)	<0.01	1.73 (1.2–2.4)	0.00	1.38 (1.0–1.9)	0.05
**Age at ART start**						
**19–24**	14.4 (12.2–16.9)		1.0		1.0	
**25–34**	11.7 (10.7–12.8)	0.58	0.80 (0.69–0.95)	0.01	0.92 (0.78–1.08)	0.32
**35–44**	11.3 (10.2–12.5)	0.63	0.79 (0.64–0.97)	0.02	0.90 (0.73–1.09)	0.28
**45–54**	10.0 (8.3–11.9)	0.38	0.69 (0.52–0.9)	0.01	0.75 (0.52–1.1)	0.12
**>55**	14.3 (10.9–18.9)	0.86	0.95 (0.67–1.35	0.78	0.89 (0.60–1.33)	0.58
**Clinic Size**						
**Large (>1,500)**	10.4 (9.7–11.2)		1.0		1.0	
**Medium (500–1499)**	9.5 (8.3–10.9)	0.29	0.72 (0.45–1.2)	0.17	0.69 (0.45–1.1)	0.08
**Small (<500 patients)**	36.0 (31.5–41.1)	0.05	2.01 (0.70–5.80)	0.20	1.63 (1.1–2.4)	0.01
**Region of the Country**						
**North Central**	8.1 (7.2–9.1)		1.0		1.0	
**North-East**	26.1 (23.0–29.6)	0.03	3.26 (1.61–6.6)	0.00	3.11 (1.7–6.0)	<0.01
**North-West**	11.2 (9.6–13.0)	0.78	0.95 (0.4–2.1)	0.91	1.08 (0.5–2.3)	0.85
**South-East**	6.2 (4.9–7.7)	0.50	0.58 (0.3–1.3)	0.18	0.54 (0.2–1.5)	0.23
**South-South**	22.8 (20.1–25.8)	<0.01	2.39 (1.4–4.1)	0.00	2.12 (1.5–3.0)	<0.01
**South-West**	9.1 (7.8–10.7)	0.23	1.13 (0.7–1.7)	0.58	1.19 (0.9–1.8)	0.28
**Baseline functional status**						
**Asymptomatic**	9.8 (9.0–10.5)		1.0		1.0	
**Symptomatic**	15.9 (14.3–17.4)	0.08	1.72 (1.3–2.3)	0.00	1.2 (1.0–1.5)	0.04
**Bedridden**	17.2 (12.7–23.4)	0.10	1.69 (0.9–3.3)	0.12	1.27 (0.8–1.9)	0.26
**Marital**						
**Single**	15.1 (13.2–17.4)		1.0		1.0	
**Married**	11.6 (10.7–12.6)	0.13	0.82 (0.7–1.0)	0.02	0.88 (0.7–1.1)	0.17
**Divorced/separated**	13.7 (10.6–17.8)	0.26	0.97 (0.7–1.3)	0.82	0.87 (0.7–1.1)	0.27
**Widowed**	10.6 (8.6–13.0)	0.09	0.74 (0.6–1.0)	0.04	0.77 (0.6–1.1)	0.10
**NRTI Backbone**						
**D4T**	9.7 (8.6–10.9)		1.0		1.0	
**TDF**	14.4 (12.7–16.3)	0.35	1.21 (0.8–1.9)	0.38	1.06 (0.8–1.4)	0.72
**AZT**	11.8 (10.9–12.8)	0.30	1.1 (0.8–1.5)	0.61	1.1 (0.8–1.4)	0.59
*Sub-optimal regimen	17.6 (11.9–26.0)	<0.01	1.61 (0.9–2.8)	0.09	2.0 (1.2–3.5)	0.01
**CTX at ART initiation**						
**Yes**	12.5 (11.8–13.4)		1.0		1.0	
**No**	8.9 (7.7–10.2)	0.37	0.7 (0.5–1.1)	0.11	1.0 (0.8–1.3)	0.90
**Year of ART start**						
**2004–2006**	9.0 (7.7–10.4)		1.0		1.0	
**2007–2009**	9.9 (9.2–10.7)	0.01	1.17 (0.8–1.7)	0.40	1.4 (1.0–1.9)	0.06
**2010–2012**	21.3 (19.2–23.6)	<0.01	2.04 (1.1–3.7)	0.02	18.3 (1.0–3.1)	0.02

^#^All variables listed in this table were included in the multivariate Competing risk proportional hazards regression model.

*Regimens not recommended in the National guidelines.

Abbreviations: CI, confidence interval; WHO, World Health Organization; Kgs, kilograms; ART, antiretroviral therapy; D4T, stavudine; 3TC, lamivudine; CTX, co-trimoxazole

**Table 4:** Patient characteristics at antiretroviral therapy initiation associated with Mortality.

		Mortality				
		Following multiple imputation (N = 3,496)				
Patient characteristics	Rate/100py (95% CI)	P values	Un-adjusted Haz. Ratio (95% CI)	P values	^#^Adjusted Haz. Ratio (95% CI)	P values
**Sex**						
**Female**	0.8 (0.6–1.1)		1.0		1.0	
**Male**	1.6 (1.1–2.2)	0.02	1.85 (1.27–2.70)	<0.01	2.06 (1.27–3.4)	0.00
**Employment Status**						
**Employ**	1.2 (1.0–1.6)		1.0		1.0	
**not currently employed**	0.8 (0.9–1.4)	0.05	0.74 (0.39–1.44)	0.38	0.64 (0.36–1.2)	0.15
**Educational status**						
**Tertiary**	0.7(0.4–1.3)		1.0		1.0	
**Secondary school**	1.3 (0.9–1.8)	0.27	1.55 (0.73–3.3)	0.25	1.63 (0.75–3.6)	0.21
**Primary school**	1.0 (0.6–2.8)	0.78	1.22 (0.49–3.0)	0.67	1.40 (0.56–3.5)	0.47
**None**	1.6 (1.0–2.8)	0.29	1.70 (0.72–4.0)	0.23	2.16 (0.81–5.8)	0.12
**CD4 at ART Start**						
**>350**	0.3 (0.07–1.1)		1.0		1.0	
**201–350**	0.4 (0.2–0.7)	<0.01	1.41 (0.25–7.9)	0.70	0.85 (0.15–4.7)	0.61
**101–200**	0.9 (0.6–1.3)	0.53	3.31 (0.70–15.7)	0.13	1.88 (0.38–9.3)	0.44
**<100**	1.9 (1.4–2.5)	<0.01	6.23 (1.35–28.7)	0.02	2.42 (0.5–11.4)	0.26
**WHO Clinical stage at ART start**						
**Stage I**	0.2 (0.1–0.4)		1.0		1.0	
**Stage II**	0.8 (0.5–1.2)	0.26	3.52 (0.98–12.6)	0.05	3.19 (0.9–11.6)	0.08
**Stage III**	1.5 (1.1–1.9)	0.01	5.61 (1.68–18.7)	0.01	4.03 (1.1–14.6)	0.03
**Stage IV**	2.1 (1.2–3.6)	0.01	7.58 (1.56–36.7)	0.01	5.68 (1.2–27.9)	0.03
**Baseline weight**						
**>60 kg**	0.6 (0.4–0.9)		1.0		1.0	
**45–60 kg**	0.9 (0.7–1.3)	0.80	1.38 (0.89–2.2)	0.15	1.04 (0.6–1.7)	0.89
**<45 kg**	2.4 (1.5–3.6)	<0.01	2.60 (1.3–5.2)	0.01	1.62 (0.8–3.2)	0.17
**Baseline hemoglobin**						
**Not anemic**	0.4 (0.2–0.8)		1.0		1	
**Mild anemia**	0.7 (0.4–1.3)	0.25	1.5 (0.61–3.6)	0.39	1.17 (0.54–2.6)	0.68
**Moderate anemia**	1.0 (0.7–1.4)	0.91	1.98 (0.89–4.4)	0.09	1.74 (0.76–4.0)	0.19
**Severe anemia**	2.2 (1.4–3.3)	<0.01	4.17 (1.6–10.7)	<0.01	3.23 (1.2–8.6)	0.02
**Age at ART start**						
**19–24**	0.9 (0.5–1.7)		1.0	1.0	1.0	
**25–34**	0.9 (0.6–1.2)	0.43	0.95 (0.56–1.6)	0.85	0.85 (0.52–1.4)	0.53
**35–44**	0.9 (0.6–1.3)	0.56	1.24 (0.6–2.4)	0.52	0.90 (0.45–1.8)	0.77
**45–54**	1.3 (0.8–2.1)	0.30	1.60 (0.68–3.77)	0.28	1.35 (0.53–3.4)	0.53
**>55**	1.7 (0.8–3.8)	0.20	1.58 (0.6–4.3)	0.38	1.03 (0.42–2.5)	0.95
**Clinic Size**						
**Large (≤1,500)**	0.6 (0.4–0.8)		1.0		1	
**Medium (500–1499)**	1.0 (0.6–1.5)	0.90	1.53 (0.47–5.0)	0.48	2.24 (0.77–6.5)	0.14
**Small (<500 patients)**	5.4 (3.9–7.7)	<0.01	3.6 (1.1–11.5)	0.03	5.88 (2.6–13.2)	0.00
**Region of the Country**						
**North Central**	1.3(1.0–1.9)		1.0		1	
**North-East**	1.2 (0.9–1.6)	0.03	0.23 (0.1–0.8)	0.02	0.08 (0.01–0.6)	0.01
**North-West**	0.2 (0.1–0.9)	0.03	0.30 (0.08–1.0)	0.05	0.36 (0.10–1.2)	0.10
**South-East**	0.4 (0.2–0.9)	0.39	0.98 (0.15–6.2)	0.99	0.70 (0.24–2.0)	0.52
**South-South**	1.9 (1.2–2.9)	0.01	0.97 (0.34–2.79)	0.96	0.66 (0.17–2.6)	0.55
**South-West**	0.6 (0.3–1.1)	0.08	0.54 (0.26–1.1)	0.1	0.53 (0.23–1.2)	0.13
**Baseline functional status**						
**Asymptomatic**	0.6 (0.4–0.8)		1.0		1.0	
**Symptomatic**	1.6 (1.2–2.2)	<0.01	2.36 (1.25–4.45)	0.01	1.45 (0.77–2.7)	0.25
**Bedridden**	2.9 (1.4–5.9)	<0.01	3.11 (1.15–8.40)	0.03	1.67 (0.71–3.9)	0.24
**Marital**						
**Single**	1.8 (1.3–2.8)		1.0		1.0	
**Married**	0.9 (0.7–1.2)	0.02	0.52 (0.29–0.93)	0.03	0.53 (0.27–1.1)	0.05
**Divorced/separated**	0.4 (0.1–3.5)	0.10	0.23 (0.05–1.02)	0.05	0.28 (0.05–1.5)	0.14
**Widowed**	1.1 (0.6–2.3)	0.10	0.66 (0.35–1.23)	0.19	0.59 (0.26–1.4)	0.23
**NRTI Backbone**						
**D4T**	0.5 (0.3–0.8)		1.0			
**TDF**	1.9 (1.3–2.6)	<0.01	2.60 (1.14–5.93)	0.02	3.17 (1.29–7.8)	0.01
**AZT**	0.9 (0.7–1.2)	0.67	1.6 (0.7–3.5)	0.29	2.10 (0.96–4.6)	0.07
*Sub-optimal regimen	1.2 (0.3–4.7)	0.82	1.96 (0.52–7.23)	0.31	2.25 (0.54–9.5)	0.27
**CTX at ART initiation**						
**Yes**	1.0(0.8–1.2)		1.0		1.0	
**No**	0.8 (0.5–1.2)	0.47	1.06 (0.53–2.1)	0.85	1.13 (0.57–2.2)	0.72
**Year of ART start**						
**2004–2006**	0.7 (0.4–1.2)		1.0		1.0	
**2007–2009**	0.8 (0.6–1.0)	0.11	0.84 (0.37–1.9)	0.69	0.60 (0.23–1.5)	0.29
**2010–2012**	1.8 (1.3–2.6)	<0.01	1.05 (0.45–2.5)	0.91	0.56 (0.23–1.4)	0.21

^#^All variables listed in this table were included in the multivariate Competing risk proportional hazards regression model.

*Regimens not recommended in the National guidelines.

Abbreviations: CI, confidence interval; WHO, World Health Organization; Kgs, kilograms; ART, antiretroviral therapy; D4T, stavudine; 3TC, lamivudine; CTX, co-trimoxazole

### ART Regimen and Co-trimoxazole Prescription at Treatment start

The vast majority (98.3%) of patients were initiated on first-line regimen. The nucleoside reverse transcriptase (NRTI) back-bone was AZT in 51.1%, D4T in 23.1% and TDF in 23.6% of the study population, and 2.1% of patients were prescribed regimens not recommended by guidelines. In original data, regimen prescription information was available for 93% (3242/3496) of patients, the majority (52.5%) of which were prescribed AZT+ 3TC+NVP/EFV, while the prescribed regimen was D4T + 3TC + NVP/EFV in 22.8% of patients; 14.4% were prescribed TDF+3TC/FTC+NVP/EFV; the least percentage (0.4%) of patients were on AZT/TDF + 3TC/FTC + LPV/r. About 9.9% of patients were prescribed other regimen. Males and females were started on essentially similar ART regimen. Co-trimoxazole (CTX) was prescribed for 80.1% (95% CI: 72.3–87.8) of patients at ART initiation.

### Retention on ART

Table 2 illustrates retention rates by duration on ART. The percentage of patients retained on ART at 1^st^, 2^nd^, 3^rd^ and 4^th^ year on ART was 81%, 74%, 67%, and 62%, respectively. Patients LTFU accounted for most attrition throughout the evaluation period.

**Fig 1:**
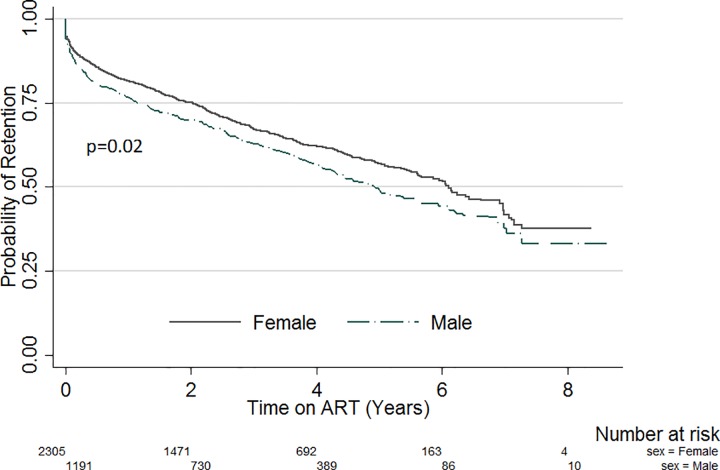
ART retention stratified by sex.

**Fig 2:**
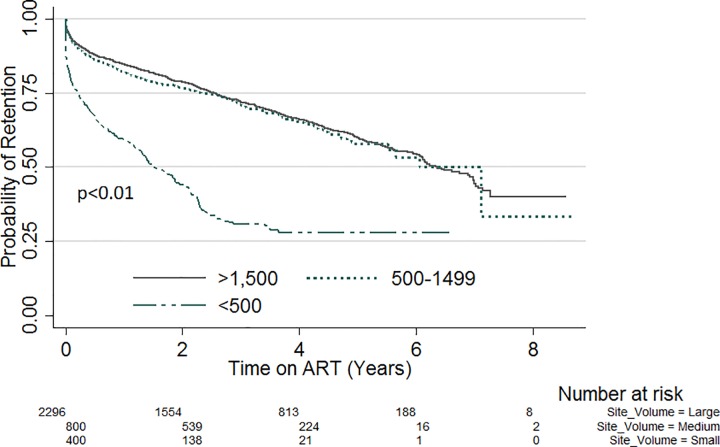
ART retention stratified by ART patient Volume.

**Fig 3:**
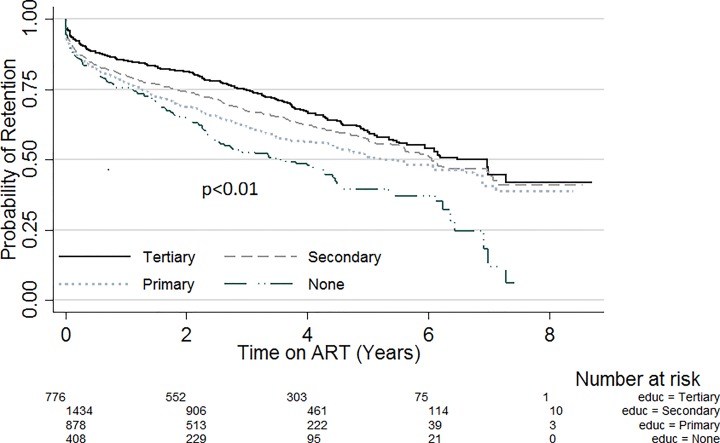
ART retention by level of education.

**Fig 4:**
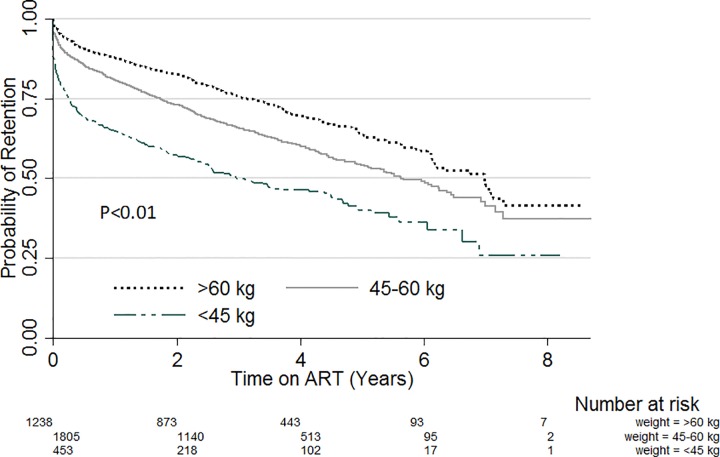
ART retention stratified by weight at ART start.

**Fig 5:**
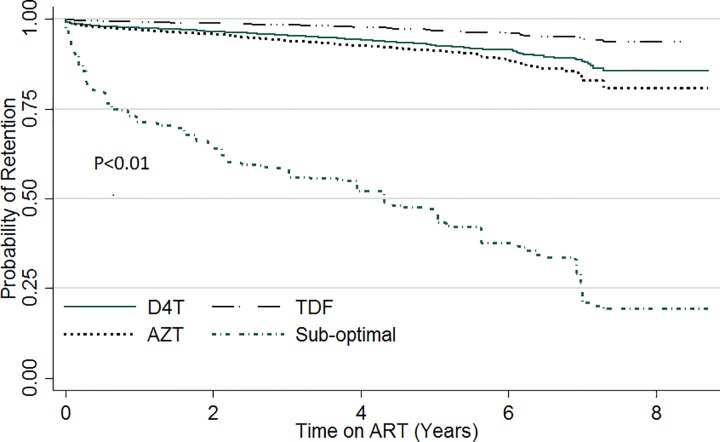
ART retention stratified by baseline regimen.

Kapler Meier curves were plotted for variables with significant variation in retention within subgroups. Fig 1 compares the probability of retention stratified by sex. Throughout the evaluation period, the probability of retention was lower amongst males compared with females (p = 0.02). Likewise, when stratified by site patient volume (Fig 2), the probability of retention was lowest in sites with patient volume less than 500 (p<0.01). There was also significant variation of retention with patient’s education status (Fig 3). Patients with no formal education had a lower probability of retention throughout the evaluation period (p<0.01). Patients with baseline weight less than 45kg were the least likely to be retained on ART throughout the review period (p<0.01) (Fig 4). When retention was stratified by ART regimen (Fig 5), patients receiving regimen that were not recommended by guidelines had the least probability of retention (p<0.01).

### Predictors of lost to follow-up and mortality

#### Sociodemographic and clinical characteristics associated with LTFU

Table 3 illustrates patient characteristics at antiretroviral therapy initiation associated with LTFU. Multivariate competing risk regression analysis revealed that patient factors predicting LTFU included: male sex (AHR = 1.36 [95% CI: 1.16 to 1.59], p<0.01); compared with patients with tertiary level education, those with primary level education (AHR = 1.41; 95% CI: 1.14 to 1.72, p<0.01), and no formal education (AHR = 1.59; 95%CI: 1.14 to 2.20, p = 0.01) were more likely to be LTFU. Predictors of LTFU also included: care in North-East (AHR = 3.11; 95% CI: 1.7 to 6.0, p<0.01) and South-South regions (AHR = 2.12; 95% CI: 1.50 to 3.0, p<0.01). Compared with large volume sites, patients receiving ART in small clinics were more likely to be LTFU (AHR = 1.63; 95% CI: 1.1 to 2.4, p = 0.01). Furthermore, compared with patients with weight >60 kg, patients with baseline weight <45kg were more likely to be LTFU (AHR = 1.65; 95% CI: 1.23 to 2.21, p<0.01). Additionally, compared with patients that were not anemic, the presence of moderate anemia (AHR = 1.46; 95% CI: 1.13 to 1.90, p = 0.01) and severe anemia were both associated with LTFU (AHR = 1.38; 95% CI: 1.0 to 1.90, p = 0.05).

#### Sociodemographic and clinical characteristics associated with mortality

Table 4 illustrates patient characteristics at antiretroviral therapy initiation associated with mortality. Patients receiving care in a small clinic had a higher risk for mortality (AHR = 5.88; 95% CI: 2.6 to 13.2, p < .01) compared with those in large volume clinics. Compared with patients receiving ART in the North-Central region, those in the North-East region of the country had significantly lower risk for mortality (AHR = 0.08; 95% CI: 0.01 to 0.6, p = 0.01). The presence of severe anemia was significantly associated with mortality (AHR = 3.23; 95% CI: 1.2 to 8.6, p = 0.02, reference: not anemic). In comparison with WHO clinical stage I disease, the risk for mortality increased with increasing WHO clinical stage: Stage II (AHR = 3.19; 95% CI: 0.9 to 11.6, p = 0.08); Stage III (AHR = 4.03; 95% CI: 1.1 to 14.6, p = 0.03) and Stage IV (AHR = 5.68; 95% CI: 1.2 to 27.9, p = 0.03). We also observed that patients initiated on TDF-containing regimen had a higher risk for mortality (AHR = 3.17; 95%CI: 1.29–7.8, P = 0.01, reference: D4T).

#### Trend of CD4-cell count and weight changes with duration on ART

The data on CD4 cell count and weight gain changes are reported for patients surviving. Table 5A illustrates changes in weighted median CD4 cell counts at follow-up stratified by sex. There was significant increase in the median CD4 cells counts over the baseline count during the study period in the study population, the median CD4 cell count was 309 (IQR: 196–449) cells/mm^3^ at 6 month and increased to 338 (IQR: 224–502) cells/mm^3^ at 12 month, 369 (IQR: IQR: 242–531) cells/mm^3^ at 24 months and 423 (IQR: 273–591) cells/mm^3^ at 36 months. This represented longitudinal increases in weighted median CD4 cell count of 148, 177, 208 and 262 at 6, 12, 24 and 36 months’ over the baseline median (161; IQR: 83–254 cells/mm^3^). Compared with males, females were more likely to start ART with higher CD4 cell counts and maintain higher counts during follow-ups. At 6 months, females had higher median CD4 cell count (317; IQR: 205–472) compared with males (288; IQR: 176–424, p = 0.003).

**Table 5:** Weight and CD4 Changes with duration on ART (weighted original data).

		A: Median CD4 Cell Counts at follow-up (Stratified by sex)						
	**Females**		**Males**		**Both sexes**			
Time on ART	**n**	**Median (IQR)**	**n**	**Median (IQR)**	P value*	**N**	**Median (IQR)**	P value*
Baseline	1891	317 (205–472)	968	288 (176–424)	<0.01	2876	309 (196–449)	
6 months	1150	366 (239–526)	533	305 (196–436)	<0.01	1683	338 (224–502)	<0.01
12 months	963	396 (263–576)	478	325 (208–451)	<0.01	1441	369 (242–531)	<0.01
24 months	774	469 (305–642)	377	372 (246–482)	<0.01	1151	423 (273–591)	<0.01
36 months	567	317 (205–472)	291	288 (176–424)	<0.01	858	309 (196–449)	<0.01
	**B: Mean weight gain over baseline (Stratified by baseline weight category)**							
	**<45kg**		**45-60kg**			**>60kg**	**Overall weight gain**	
Time on ART	**n**	**Mean (95%CI)**	**n**	**Mean (95%CI)**	**n**	**Mean (95%CI)**	**N**	**Mean (95%CI)**
6 months	267	7.4kg (6.5–8.4)	1343	3.2kg (2.7–3.7)	907	1.4kg (0.6–2.3)	2517	3.0 (2.5–3.6)
12 months	236	10.0kg (9.0–11.0)	1254	5.3kg (4.6–5.9)	889	3.1kg (2.2–4.0)	2379	4.9 (4.3–5.6)
24 months	191	11.1kg (9.9–12.3)	1019	6.3kg (5.5–7.1)	746	4.1kg (3.1–5.1)	1956	5.9 (5.2–6.7)
36 months	149	11.7kg (10.5–12.9)	757	6.8kg (6.0–7.6)	565	4.2kg (3.2–5.2)	1471	6.3 (5.5–7.0)

Abbreviations: CI, confidence interval; IQR, interquartile range; Kgs, kilograms, Tx, Treatment.

*Analysis was done only for surviving patients and those with available CD4 count.

Table 5B illustrates mean weight gain in the study population with time on ART. Overall, the greatest weight gain of 3kg (95%CI: 2.5–3.6) over baseline weight occurred in the first 6 months on ART and increased by a range of 1-2kg at 12, 24, and 36 months. Patients with baseline weight <45kg had the most increase in mean weight in the first six months on ART with an increase of 7.4kg compared with other baseline weight categories that had between 1 and 3 kg gains in the same period. However, the observed weight gains by baseline weight categories were not statistically significant.

## Discussion

This is the first national representative ART study in Nigeria and demonstrates very important findings from the Nigerian ART program. The observed LTFU, mortality and overall attrition rates in this study were similar to those reported for several countries in sub-Sahara Africa [55–57]. In addition, there was significant variation in retention by site’s patient volume and across the six regions in Nigeria. High and medium volume sites generally had better retention rates, while sites in the North-East and South-south regions, two regions that have witnessed significant communal strife and terrorism had the least retention rates. Our findings suggest that low volume sites and sites in North-East and South-South region will require significant attention to improve retention rates. Additionally, consistent with previous reports [18, 19, 58, 59], we found that males were least likely to be retained on ART compared with females, suggesting that male-specific interventions are needed to improve retention rates. We also observed that the risk for LFTU which was the major driver of attrition in our study was higher among patients with moderate to severe anemia, symptomatic functional status and those with low baseline weights. Furthermore, the risk for mortality increased with increasing WHO clinical staging suggesting that earlier initiation on ART may improve program outcome.

As reported by a similar study in Mozambique [58], we also found that LTFU rates generally increased with program expansion. The cohorts initiated on ART in 2010–2012 had higher LTFU rates compared with the 2004–2006 cohorts. Further research to better understand the reason for poorer outcomes during program expansion is required. However, the significant expansion to small-volume sites between 2010 and 2012 may be a factor. Lastly, the observed mortality rate of 1.1/100py in our study was much lower than those previously reported in similar settings [2, 58, 60]. Previous studies in resource-constrained settings have suggested that majority of patients that were LTFU may have actually died [61–63]. Hence, it is likely that our mortality results may be an underestimation.

Studies in resource-constrained settings have specifically identified the first 3 to 6 months of therapy as the period with the highest death rates [64–66]. The primary factor associated with high early mortality was identified as the advanced immunodeficiency of those starting ART, including low baseline CD4 cell counts (particularly <50 cells/mm³), high prevalence of co-infections and more advanced clinical stage (III and IV) as well as malnutrition (defined by BMI of < 18) and anemia (hemoglobin < 10 g/dl) [67–69]. A collaborative analysis of prospective studies reported median CD4 cell counts at baseline of 102, 213, and 172 cells/ mm^3^ in South Africa, Europe, and North America, respectively[70]. The study reported higher early mortality after starting ART among the South African cohort [70]. Additionally, early mortality was reported mainly in patients starting ART with CD4 cell count <50 cells/ mm^3^. Our study observed a median CD4 cell count was 161 cells/mm^3^ and found that the highest death rates occurred within the first three months on ART. We also found that mortality rates were higher in patients with severe anemia and in those with baseline weight less than 45kg. Additionally, compared with patients with CD4 cell count <350 cells/mm^3^, those with CD4 cell count <100 cells/mm^3^ had a higher mortality rates. Furthermore, we observed that the risk for mortality increased with increasing WHO staging. Our findings corroborate previous reports that mortality will likely be highest among patients with severe immunosuppression [70].

We found that persons receiving ART in a small clinic were more likely to be LTFU or die compared with those receiving ART in large clinics. The fact that large clinics in Nigeria tend to be in secondary or tertiary health facilities with specialist care, while small clinics are usually primary health centers manned by nurses and community health extension workers, suggests that this finding may be due more to the quality of care received rather than the size of the clinic. However, a systematic review of several studies suggested that task shifting from doctors to nurses or from health care professionals to lay health workers can potentially reduce costs of ART provision without compromising health outcomes for patients[71]. Another possible explanation is the fact that there was less patient volume in smaller clinics, meaning that health workers at these clinics had less clinical experience than the busier clinics. These findings suggest that the exact reasons for higher LTFU and mortality in small clinics in our study require further investigation.

LTFU accounted for the majority of all attrition among patients on ART during the evaluation period, this observation was similar with reports from previous studies in Nigeria [17, 19] and findings from other resource-limited settings [19, 61, 72]. Additionally, higher proportions of females were retained on ART compared with males, possibly due to gender differences in health-seeking behavior [73]. These observations highlight the need to develop programs that will improve uptake of services, particularly among men.

Another interesting finding in our study was the higher risk for mortality observed among patients on TDF-containing regimen, adjusting for the effects of baseline clinical factors including baseline CD4 cell counts, WHO stage, baseline hemoglobin concentration and weights. The finding was consistent with that reported in a similar study in Nigeria among patients receiving ART [17]. A similar study in Uganda among over five thousand treatment-naive patients starting ART observed higher mortality among those initiated on TDF-containing regimen[74]. These findings require more elaborate research because both randomized and observational studies have reported sustained viral suppression and low prevalence of nephrotoxity with use of TDF [75–77]. However, we found no association between LTFU and TDF use. The exact reasons for higher risk for mortality among patients on TDF-containing regimen in our study require further investigations.

We found that persons with primary or no formal education were more likely to be LTFU. This finding was consistent with those reported by similar studies from Nigeria [78]. This may be because individuals with low or no formal education are less likely to understand the information provided about their illness and treatment course in English. One study on outcomes and reasons for LTFU among HIV-infected women reported that inability to understand the information provided was a reason for LTFU [79]. This finding suggests that providing educational materials in local languages may contribute to improved retention.

Our study found both the median CD4 cell counts and weight showed improvements in the follow-up periods. Increased CD4 cell count is a key marker of immune restoration [80–83]. Vigorous improvements in CD4 cell levels following ART initiation have been reported in both clinical trials and observational studies from high resource countries [84–86]. Among studies reporting on early outcomes from ART expansion, Coetzee et al noted an increase of 245 cells from the median baseline of 43/ml cells after 24 months on ART at a South African site [87], and Ferradini, 233 cells in Cambodia from a median baseline of 11/ml cells for the same time period [88]. One study reported median increases in CD4 cell counts were 114, 181, and 248 cells/mm3 at 6, 12, and 24 months of HAART, respectively[89]. The observed longitudinal increases in CD4 cell count over baseline of +147, +181, +210 and +259 at 6, 12, 24 and 36 months’ respectively were comparable with those reported by previous cohort studies in low-resource settings[89, 90], and suggests progressive improvement in immune response. The changes in CD4 cell count and weight gains observed in our study must be taken with caution due to substantial missing data for both variables during. However, when there were missing data, the missingness was not consistently associated with changes in geographic area or clinic size. Additionally, analysis of changes in follow-up CD4 cell count and weight were done only on surviving patients. Our data suggest that the highest risk for mortality or LTFU were among patients with very low baseline weights or CD4 cell count. Hence, the most immunosuppressed patients may not have participated in follow-up analysis.

The large number of patients studied and the wide geographic spread makes for reliable inferences. A limitation of our study was the inadequate facility data providing information on factors influencing client retention, for example, informal transfers, adherence to ART, patient waiting time, and quality of service delivery. In addition, our analyses were based on routinely collected data, which were incomplete for some baseline and follow-up variables. Additionally, the study was conducted in PEPFAR funded sites. At the time the study was conducted, PEPFAR supported sites accounted for the majority of ART sites in Nigeria. Nevertheless, there are likely program peculiarities that may make study sites differ in patient outcomes from sites that are not funded by PEPFAR. Furthermore, since we could not establish the true status of patients LTFU, our mortality estimate is likely an under-estimate and the LTFU estimate an over-estimate.

## Conclusion

There was progressive immune recovery among patients on ART in Nigeria. The percentage of patients alive and on ART after four years on ART was 61.7%. LTFU accounted for the majority of attrition. Attrition rates were comparable with rates from similar settings. Factors predicting LTFU included: Male sex, Primary or no formal education status, and care in North-East and South-South regions, moderate/severe anemia and baseline weight <45kg. Retention interventions targeting men and those with lower levels of education are needed. Further research to understand geographic and clinic size variations in attrition rates is warranted.

## Supporting Information

S1 File Nigeria ART Evaluation Data Abstraction Tool Final 19 Nov 2012. (DOC)

S2 File Minimal dataset. (DTA)
