# Correction: Outcomes of Nigeria's HIV/AIDS Treatment Program for Patients Initiated on Antiretroviral Treatment between 2004-2012

**DOI:** 10.1371/journal.pone.0170912

**Published:** 2017-01-23

**Authors:** Ibrahim Dalhatu, Dennis Onotu, Solomon Odafe, Oseni Abiri, Henry Debem, Simon Agolory, Ray W. Shiraishi, Andrew F. Auld, Mahesh Swaminathan, Kainne Dokubo, Evelyn Ngige, Chukwuemeka Asadu, Emmanuel Abatta, Tedd V. Ellerbrock

The affiliation for the tenth author is incorrect. Kainne Dokubo is not affiliated with #2 but with #3 Division of Global HIV/AIDS, Center for Global Health, U.S. Centers for Disease Control & Prevention, Atlanta, Georgia, United States of America.

## References

[1] Dalhatu I, Onotu D, Odafe S, Abiri O, Debem H, Agolory S, et al (2016) Outcomes of Nigeria's HIV/AIDS Treatment Program for Patients Initiated on Antiretroviral Treatment between 2004–2012. PLoS ONE 11(11): e0165528 doi:10.1371/journal.pone.0165528 27829033 10.1371/journal.pone.0165528 PMC5102414

