# Supplementary material for: Outcomes of Nigeria's HIV/AIDS Treatment Program for Patients Initiated on Antiretroviral Treatment between 2004-2012
Source: PLoS One. 2016 Nov 9;11(11):e0165528. doi: 10.1371/journal.pone.0165528 (PMC5102414; doi:10.1371/journal.pone.0165528)
Supplement: S1 File — (DOC) [file pone.0165528.s001.doc]

**Adult Study ID:A __ __ __ Name of Hospital/Clinic: _____________________**

**Abstractor’s Name: ________________________ Date of Abstraction: D D / M M / Y Y**

| **A. PERSONAL INFORMATION** | | | | | | | | | | | | | | | | | | | | | | | | |
| --- | --- | --- | --- | --- | --- | --- | --- | --- | --- | --- | --- | --- | --- | --- | --- | --- | --- | --- | --- | --- | --- | --- | --- | --- |
| 1. Sex: | | | | | | | **Male**  **Female** | | | | | | | | | | | | | | | | | |
| 2. Date of Birth: | | | | | | | D D **/** M M **/** Y Y \ | | | | | | | | | | | | | | | | | |
| 3. Age at and date of enrollment in ART program: | | | | | | | D D **/** M M **/** Y Y **____Years** (cannot be missing) | | | | | | | | | | | | | | | | | |
| 4. Marital status of patient at the time of enrollment in ART program: | | | | | | | **Single**  **Married**  **Divorced**  **Widowed**  **Other, specify: _______**  **Missing** | | | | | | | | | | | | | | | | | |
| 5. Partner/spouse HIV status (through the most recent visit): | | | | | | | **HIV positive**  **HIV negative**  **Missing/Unknown** | | | | | | | | | | | | | | | | | |
| 6. Patient education level at time of enrollment in ART program: | | | | | | | **None**  **Primary school**  **Secondary school**  **Post Secondary**  **University**  **Other, specify: ___________**  **Missing** | | | | | | | | | | | | | | | | | |
| 7. Patient employment status at the time of enrollment in ART program: | | | | | | | **Employed**  **No, not currently employed**  **Missing** | | | | | | | | | | | | | | | | | |
| 8. Was patient pregnant at time of enrollment in ART program? | | | | | | | **Yes**  **No**  **N/A (male)**  **Missing** | | | | | | | | | | | | | | | | | |
| 9. Did patient start ART at a different clinic BEFORE transferring into this clinic? | | | | | | | **No** *(skip to question 12)* **Yes,**  **if yes: *date of transfer in:*____/_____/_______** **Missing** | | | | | | | | | | | | | | | | | |
| 10. If patient is a “**transfer in**”, enter the dates of HIV ART initiation at previous facility *(if available)*. | | | | | | | **ART Initiation:** D D **/** M M **/** Y Y  **Missing** | | | | | | | | | | | | | | | | | |
| 11. Name of the ART regimen initiated at previous facility? | | | | | | | **_______________/__________________/________________** **Missing** | | | | | | | | | | | | | | | | | |
| **B. CLINICAL INFORMATION** | | | | | | | | | | | | | | | | | | | | | | | | |
| 12. Date of first confirmed HIV Positive test: | | | | | | | D D **/** M M **/** Y Y  **Missing** | | | | | | | | | | | | | | | | | |
| 13. HIV type: | | | | | | | **HIV1** **HIV2** **HIV1 and 2**  **Type unknown** **Missing** | | | | | | | | | | | | | | | | | |
| 14. Patient height: | | | | | | | **_____ Meters**  **Missing** | | | | | | | | | | | | | | | | | |
| 15. Patient weight at start of ART: | | | | | | | **__________ kg**  **Missing** | | | | | | | | | | | | | | | | | |
| 16. Date medically eligible for ART: | | | | | | | D D **/** M M **/** Y Y  **Missing** | | | | | | | | | | | | | | | | | |
| 17. Date ART started: | | | | | | | D D **/** M M **/** Y Y (cannot be missing) | | | | | | | | | | | | | | | | | |
| 18. Eligibility Criteria for ART: *(check all that apply)* | | | | | | | **Clinically only**  **CD4**  **Total Lymphocytes Count**  **Missing**  **Other, specify_______________** | | | | | | | | | | | | | | | | | |
| 19. CD4 at start of ART: | | | | | | | **_____ cells/mm^3^ *date of test,*** D D **/** M M **/** Y Y **Missing** | | | | | | | | | | | | | | | | | |
| 20. Clinical stage at start of ART: | | | | | | | **Stage I**  **Stage II**  **Stage III**  **Stage VI**  **Missing** | | | | | | | | | | | | | | | | | |
| 21. Functional status at start of ART: | | | | | | | **Asymptomatic (working)**  **Symptomatic normal activity (ambulatory)**  **Bed ridden <50% of day in last month**  **Bed ridden >50% of day in last month**  **Missing** | | | | | | | | | | | | | | | | | |
| 22. TB status at start of ART: | | | | | | | **No TB**  **On INH prophylaxis**  **Suspected TB**  **Prior history of TB treatment**  **On TB treatment**  **Missing** | | | | | | | | | | | | | | | | | |
| 23. Opportunistic Infections (OIs) **during** ART treatment and dates of diagnosis (dx): *(check all that apply)* | | | | | | | **Chronic diarrhea**  **Tuberculosis (pulm/extrapulm)**  **PCP**  **Cryptococcosis**  **Kaposi Sarcoma**  **Herpes Zoster**  **Vaginal thrush**  **Herpes Simplex**  **Other,specify:**  **No documented OIs** | | | | | | | | | | | ***date of dx,*** D D **/** M M **/** Y Y  ***date of dx,*** D D **/** M M **/** Y Y  ***date of dx,*** D D **/** M M **/** Y Y  ***date of dx,*** D D **/** M M **/** Y Y  ***date of dx,*** D D **/** M M **/** Y Y  ***date of dx,*** D D **/** M M **/** Y Y  ***date of dx,*** D D **/** M M **/** Y Y  ***date of dx,*** D D **/** M M **/** Y Y  ***date of dx,*** D D **/** M M **/** Y Y | | | | | | |
| 24. History of sexually transmitted infections before or during ART treatment: *(check all that apply)* | | | | | | | **Gonorrhea**  **Chlamydia**  **Syphilis**  **Herpes**  **Other (s), specify_______________**  **No history of sexually transmitted infection** | | | | | | | | | | | | | | | | | |
| 25. History of other chronic illnesses e.g. Diabetes, Kidney disease, etc before or during ART treatment: | | | | | | | **Yes**  **No**  **Missing**  **If yes, please specify: 1. _____________2.__ __________3. ________** | | | | | | | | | | | | | | | | | |
| 26. Was Patient on Cotrimoxazole (CTX) at start of ART? | | | | | | | **Yes,**  **No**  **Missing** | | | | | | | | | | | | | | | | | |
| 27. Was Patient on Cotrimoxazole (CTX) at last visit? | | | | | | | **Yes,**  **No**  **Missing** | | | | | | | | | | | | | | | | | |
| **C. ART REGIMEN** | | | | | | | | | | | | | | | | | | | | | | | | |
| 28. If a drug is changed in a regimen (substitution) or if the whole regimen is changed (switch), give date of the change, old and new regimens, and reason(s) for change. Use the below list to pick the reason (s) for change (*complete this section only if there is change in ART regimen, if no change, select no change #7 below*):  **1 = Toxicity 2 = pregnancy 3 = anemia 4 = active TB**  **5 = new medicine available 6 = break in supply of a drug 7 = other, please specify**  **If documentation shows no regimen changes, tick here:**  **NO REGIMEN CHANGES** | | | | | | | | | | | | | | | | | | | | | | | | |
| **Date of change** | | | | | **Old Regimen** | | | | **New Regimen** | | | | | | **Reason(s) for change** | | | | **(enter appropriate ART 3 letters/letter + number from below)**  1a**.**d4t-3TC-NVP  1b. d4t-3TC-EFV  1c. TDF-3TC-NVP  1d. TDF-3TC-EFV  1e. TDF-FTC-NVP  1f. TDF-FTC-EFV  1g. AZT-3TC-NVP  1h. AZT-3TC-EFV  1j AZT-FTC-NVP  1k. AZT-FTC-EFV  2a**.**ABC-ddI-SQV/r  2b. TDF-ddI-IDV/r  2c. TDF-3TC-LPV/r  2d. TDF-3TC-IDV/r  2e. TDF-3TC-SQV/r  2f. TDF-FTC-LPV/r  2g. TDF-FTC-IDV/r  2h. TDF-FTC-SQV/r  2i. AZT-TDF-3TC-LPV/r  2j. AZT-TDF-3TC  2k. AZT-FTC-TDF-LPV/r  3a. TDF-ddI-IDV/r  3b. TDF-ddI-LPV/r  3c. ABC-ddI-SQV/r  3d. ABC-ddI-LPV/r  44. Other, specify: / / | | | | | |
| D D **/** M M **/** Y Y | | | | |  | | | |  | | | | | |  | | | |
| D D **/** M M **/** Y Y | | | | |  | | | |  | | | | | |  | | | |
| D D **/** M M **/** Y Y | | | | |  | | | |  | | | | | |  | | | |
| D D **/** M M **/** Y Y | | | | |  | | | |  | | | | | |  | | | |
| D D **/** M M **/** Y Y | | | | |  | | | |  | | | | | |  | | | |
| D D **/** M M **/** Y Y | | | | |  | | | |  | | | | | |  | | | |
| D D **/** M M **/** Y Y | | | | |  | | | |  | | | | | |  | | | |
| D D **/** M M **/** Y Y | | | | |  | | | |  | | | | | |  | | | |
| D D **/** M M **/** Y Y | | | | |  | | | |  | | | | | |  | | | |
| D D **/** M M **/** Y Y | | | | |  | | | |  | | | | | |  | | | |
| D D **/** M M **/** Y Y | | | | |  | | | |  | | | | | |  | | | |
| D D **/** M M **/** Y Y | | | | |  | | | |  | | | | | |  | | | |
| D D **/** M M **/** Y Y | | | | |  | | | |  | | | | | |  | | | |
| D D **/** M M **/** Y Y | | | | |  | | | |  | | | | | |  | | | |
| D D **/** M M **/** Y Y | | | | |  | | | |  | | | | | |  | | | |
| D D **/** M M **/** Y Y | | | | |  | | | |  | | | | | |  | | | |
| D D **/** M M **/** Y Y | | | | |  | | | |  | | | | | |  | | | |
| D D **/** M M **/** Y Y | | | | |  | | | |  | | | | | |  | | | |
| D D **/** M M **/** Y Y | | | | |  | | | |  | | | | | |  | | | |
| D D **/** M M **/** Y Y | | | | |  | | | |  | | | | | |  | | | |
| D D **/** M M **/** Y Y | | | | |  | | | |  | | | | | |  | | | |
| D D **/** M M **/** Y Y | | | | |  | | | |  | | | | | |  | | | |
| D D **/** M M **/** Y Y | | | | |  | | | |  | | | | | |  | | | |
| D D **/** M M **/** Y Y | | | | |  | | | |  | | | | | |  | | | |
| D D **/** M M **/** Y Y | | | | |  | | | |  | | | | | |  | | | |
| D D **/** M M **/** Y Y | | | | |  | | | |  | | | | | |  | | | |
| D D **/** M M **/** Y Y | | | | |  | | | |  | | | | | |  | | | |
| D D **/** M M **/** Y Y | | | | |  | | | |  | | | | | |  | | | |
| D D **/** M M **/** Y Y | | | | |  | | | |  | | | | | |  | | | |
| D D **/** M M **/** Y Y | | | | |  | | | |  | | | | | |  | | | |
| D D **/** M M **/** Y Y | | | | |  | | | |  | | | | | |  | | | |
| **D. ART INTERRUPTIONS AND ADVERSE EVENTS** | | | | | | | | | | | | | | | | | | | | | | | | |
| 29a. Any history of Stopping ART use? | | | | | | | **If yes, enter 1^st^ stop date:** D D **/** M M **/** Y Y  **If yes, enter 1^st^ restart date:** D D **/** M M **/** Y Y  **If stopped a 2^nd^ time, enter 2^nd^ stop date:** D D **/** M M **/** Y Y  **If stopped a 2^nd^ time, enter 2^nd^ start date:** D D **/** M M **/** Y Y  **If stopped a 3^rd^ time, enter 3^rd^ stop date:** D D **/** M M **/** Y Y  **If stopped a 3^rd^ time, enter 3^rd^ start date:** D D **/** M M **/** Y Y  **No – No history of stopping ART use** , (*skip to question 30*) | | | | | | | | | | | | | | | | | |
| | 29b. If patient **stopped ART**, what was the reason? (*Check all that apply)* | **Developed Active TB**  **Drug toxicity/intolerance**  **IRIS**  **Pregnancy**  **Other, _______________**  **Unknown** | D D **/** M M **/** Y Y  D D **/** M M **/** Y Y  D D **/** M M **/** Y Y  D D **/** M M **/** Y Y  D D **/** M M **/** Y Y | | --- | --- | --- | | | | | | | | | | | | | | | | | | | | | | | | | | | | | | |
| 30. Any documented adverse events to ART that patient developed? | | | | | | | **Yes**  **No**  ***If Yes, check all that apply*:**  **Severe rash**  **IRIS**  **Anemia**  **Hepatitis**  **Neuropathy**  **Other, specify:______________________** | | | | | | | | | | | | | | | | | |
| **E. FOLLOW UP STATUS** | | | | | | | | | | | | | | | | | | | | | | | | |
| 31. List the dates of **ALL** scheduled and actual clinic visits starting from the date patient was started on ART and enter weights, follow-up status for each prior visit (*use page 7 if more space is needed*)  For follow up status use the following codes: **1 = On treatment 2 = Dead 3 = Stopped ART**  **4 = Lost to follow up 5 = Transferred out 6 = Restarted ART 7 = Other, specify____**  **8 = Missing** | | | | | | | | | | | | | | | | | | | | | | | | |
| **Scheduled date** | | | | **Actual Visit Date** | | | | | | | | **Weight** | | | | | | | | | **Follow up status** | | | |
| D D **/** M M **/** Y Y | | | | D D **/** M M **/** Y Y | | | | | | | | **___Kg**  **Missing** | | | | | | | | |  | | | |
| D D **/** M M **/** Y Y | | | | D D **/** M M **/** Y Y | | | | | | | | **___Kg**  **Missing** | | | | | | | | |  | | | |
| D D **/** M M **/** Y Y | | | | D D **/** M M **/** Y Y | | | | | | | | **___Kg**  **Missing** | | | | | | | | |  | | | |
| D D **/** M M **/** Y Y | | | | D D **/** M M **/** Y Y | | | | | | | | **___Kg**  **Missing** | | | | | | | | |  | | | |
| D D **/** M M **/** Y Y | | | | D D **/** M M **/** Y Y | | | | | | | | **___Kg**  **Missing** | | | | | | | | |  | | | |
| D D **/** M M **/** Y Y | | | | D D **/** M M **/** Y Y | | | | | | | | **___Kg**  **Missing** | | | | | | | | |  | | | |
| D D **/** M M **/** Y Y | | | | D D **/** M M **/** Y Y | | | | | | | | **___Kg**  **Missing** | | | | | | | | |  | | | |
| D D **/** M M **/** Y Y | | | | D D **/** M M **/** Y Y | | | | | | | | **___Kg**  **Missing** | | | | | | | | |  | | | |
| D D **/** M M **/** Y Y | | | | D D **/** M M **/** Y Y | | | | | | | | **___Kg**  **Missing** | | | | | | | | |  | | | |
| D D **/** M M **/** Y Y | | | | D D **/** M M **/** Y Y | | | | | | | | **___Kg**  **Missing** | | | | | | | | |  | | | |
| D D **/** M M **/** Y Y | | | | D D **/** M M **/** Y Y | | | | | | | | **___Kg**  **Missing** | | | | | | | | |  | | | |
| D D **/** M M **/** Y Y | | | | D D **/** M M **/** Y Y | | | | | | | | **___Kg**  **Missing** | | | | | | | | |  | | | |
| D D **/** M M **/** Y Y | | | | D D **/** M M **/** Y Y | | | | | | | | **___Kg**  **Missing** | | | | | | | | |  | | | |
| D D **/** M M **/** Y Y | | | | D D **/** M M **/** Y Y | | | | | | | | **___Kg**  **Missing** | | | | | | | | |  | | | |
| **F. COUNSELLING & SUPPORT SERVICES** | | | | | | | | | | | | | | | | | | | | | | | | |
| **Did Patient Receive any of the following?** | | | | | | | | | | | | | | | | | | | | | | | | |
| 32. Pre-ART counseling | | | | | | | **Yes, *No of times*____**  **No**  **Not collected**  **Missing** | | | | | | | | | | | | | | | | | |
| 33. Counseling at ART initiation | | | | | | | **Yes, *No of times*____**  **No**  **Not collected**  **Missing** | | | | | | | | | | | | | | | | | |
| 34. Any adherent counseling during follow-up | | | | | | | **Yes, *No of times*____**  **No**  **Not collected**  **Missing** | | | | | | | | | | | | | | | | | |
| 35. Does patient attend a support Group? | | | | | | | **Yes**  **No**  **Missing**  **If yes, please specify:__________________________** | | | | | | | | | | | | | | | | | |
| 36. Support services used by the patient since initiation of ART (*check all that apply*) | | | | | | | **Home based care**  **Nutritional support**  **Community based support groups**  **Other, please specify:________________**  **Not using any support services**  **Missing** | | | | | | | | | | | | | | | | | |
| 37. Does patient use condoms? | | | | | | | | **Always**  **most of the time**  **Occasionally**  **Do not use condoms**  **Not sexually active**  **Not assessed**  **Missing** | | | | | | | | | | | | | | | | |
| **G. PHARMACY REGISTER** | | | | | | | | | | | | | | | | | | | | | | | | |
| 38. List dates of **ALL** ARV collected, please record date refill given, ARV regimen (use ARV codes from question 28), and number of days for which ARV prescription is given (*use page 8 if more space is needed*) | | | | | | | | | | | | | | | | | | | | | | | | |
| Date | | | Regimen | | | | | | | # days of prescription | | | | Date | | | Regimen | | | | | | | # days of prescription |
| D D **/** M M **/** Y Y | | |  | | | | | | |  | | | | D D **/** M M **/** Y Y | | |  | | | | | | |  |
| D D **/** M M **/** Y Y | | |  | | | | | | |  | | | | D D **/** M M **/** Y Y | | |  | | | | | | |  |
| D D **/** M M **/** Y Y | | |  | | | | | | |  | | | | D D **/** M M **/** Y Y | | |  | | | | | | |  |
| D D **/** M M **/** Y Y | | |  | | | | | | |  | | | | D D **/** M M **/** Y Y | | |  | | | | | | |  |
| D D **/** M M **/** Y Y | | |  | | | | | | |  | | | | D D **/** M M **/** Y Y | | |  | | | | | | |  |
| D D **/** M M **/** Y Y | | |  | | | | | | |  | | | | D D **/** M M **/** Y Y | | |  | | | | | | |  |
| D D **/** M M **/** Y Y | | |  | | | | | | |  | | | | D D **/** M M **/** Y Y | | |  | | | | | | |  |
| D D **/** M M **/** Y Y | | |  | | | | | | |  | | | | D D **/** M M **/** Y Y | | |  | | | | | | |  |
| D D **/** M M **/** Y Y | | |  | | | | | | |  | | | | D D **/** M M **/** Y Y | | |  | | | | | | |  |
| D D **/** M M **/** Y Y | | |  | | | | | | |  | | | | D D **/** M M **/** Y Y | | |  | | | | | | |  |
| **H. LABORTORY RESULTS** | | | | | | | | | | | | | | | | | | | | | | | | |
| 39. Please record the test dates and values of **ALL** CD4 **cells/mm^3^** counts, viral loads (VL) **Copies/dl**, Hemoglobin (Hgb) **g/dl** levels, Alanine aminotransferase (ALT) **U/I**, and Creatinine (Cr) umol/L, for this patient (*use page 9 if more space is needed*) | | | | | | | | | | | | | | | | | | | | | | | | |
| **Visit #** | **CD4** | **Date** | | | | **VL** | **Date** | | | | **Hgb** | | **Date** | | | **ALT U/I** | | | | **Date** | | **Cr** | **Date** | |
| 1 |  | DD**/**MM**/**YY | | | |  | DD**/**MM**/**YY | | | |  | | DD**/**MM**/**YY | | |  | | | | DD**/**MM**/**YY | |  | DD**/**MM**/**YY | |
| 2 |  | DD**/**MM**/**YY | | | |  | DD**/**MM**/**YY | | | |  | | DD**/**MM**/**YY | | |  | | | | DD**/**MM**/**YY | |  | DD**/**MM**/**YY | |
| 3 |  | DD**/**MM**/**YY | | | |  | DD**/**MM**/**YY | | | |  | | DD**/**MM**/**YY | | |  | | | | DD**/**MM**/**YY | |  | DD**/**MM**/**YY | |
| 4 |  | DD**/**MM**/**YY | | | |  | DD**/**MM**/**YY | | | |  | | DD**/**MM**/**YY | | |  | | | | DD**/**MM**/**YY | |  | DD**/**MM**/**YY | |
| 5 |  | DD**/**MM**/**YY | | | |  | DD**/**MM**/**YY | | | |  | | DD**/**MM**/**YY | | |  | | | | DD**/**MM**/**YY | |  | DD**/**MM**/**YY | |
| 6 |  | DD**/**MM**/**YY | | | |  | DD**/**MM**/**YY | | | |  | | DD**/**MM**/**YY | | |  | | | | DD**/**MM**/**YY | |  | DD**/**MM**/**YY | |
| 7 |  | DD**/**MM**/**YY | | | |  | DD**/**MM**/**YY | | | |  | | DD**/**MM**/**YY | | |  | | | | DD**/**MM**/**YY | |  | DD**/**MM**/**YY | |
| 8 |  | DD**/**MM**/**YY | | | |  | DD**/**MM**/**YY | | | |  | | DD**/**MM**/**YY | | |  | | | | DD**/**MM**/**YY | |  | DD**/**MM**/**YY | |
| 9 |  | DD**/**MM**/**YY | | | |  | DD**/**MM**/**YY | | | |  | | DD**/**MM**/**YY | | |  | | | | DD**/**MM**/**YY | |  | DD**/**MM**/**YY | |
| 10 |  | DD**/**MM**/**YY | | | |  | DD**/**MM**/**YY | | | |  | | DD**/**MM**/**YY | | |  | | | | DD**/**MM**/**YY | |  | DD**/**MM**/**YY | |
| 11 |  | DD**/**MM**/**YY | | | |  | DD**/**MM**/**YY | | | |  | | DD**/**MM**/**YY | | |  | | | | DD**/**MM**/**YY | |  | DD**/**MM**/**YY | |
| 12 |  | DD**/**MM**/**YY | | | |  | DD**/**MM**/**YY | | | |  | | DD**/**MM**/**YY | | |  | | | | DD**/**MM**/**YY | |  | DD**/**MM**/**YY | |
| **40**. Hepatitis B status for this patient is: | | | | | | | | **Antibodies Positive**  **Antigen positive**  **Negative**  **Unknown**  **Missing** | | | | | | | | | | | | | | | | |
| **41**. Hepatitis C status for this patient: | | | | | | | | **Positive**  **Negative**  **Unknown**  **Missing**  **Date of test:**  D D **/** M M **/** Y Y | | | | | | | | | | | | | | | | |
| 1. **KEY OUTCOMES** | | | | | | | | | | | | | | | | | | | | | | | | |
| **42. Date of last clinic visit:** | | | | | | | | D D **/** M M **/** Y Y | | | | | | | | | | | | | | | | |
| 43. Patient’s outcome at the last visit? | | | | | | | | **Died, *date of death:*** *D D* ***/*** *M M* ***/*** *Y Y*  **Alive, on ART**  **Transferred out, *date of transfer out:*** *D D* ***/*** *M M* ***/*** *Y Y*  **Stopped ART, *date of voluntarily stopping care:*** *D D* ***/*** *M M* ***/*** *Y Y*  **Lost to follow up** | | | | | | | | | | | | | | | | |
| 44. If patient died, what was the documented cause of death? | | | | | | | | **Pneumonia (not P TB / PCP)**  **Cryptococcal meningitis**  **Acute diarrhoea**  **Chronic diarrhoea**  **PTB**  **EPTB**  **Other, specify:______________**  **Unknown**  **Missing** | | | | | | | | | | | | | | | | |

If additional space is needed for **question 32,** please use the space below

| *Continuation from page 4, question 32* | | | |
| --- | --- | --- | --- |
| **Scheduled date** | **Actual Visit Date** | **Weight** | **Follow up status** |
| D D **/** M M **/** Y Y | D D **/** M M **/** Y Y | **___Kg**  **Missing** |  |
| D D **/** M M **/** Y Y | D D **/** M M **/** Y Y | **___Kg**  **Missing** |  |
| D D **/** M M **/** Y Y | D D **/** M M **/** Y Y | **___Kg**  **Missing** |  |
| D D **/** M M **/** Y Y | D D **/** M M **/** Y Y | **___Kg**  **Missing** |  |
| D D **/** M M **/** Y Y | D D **/** M M **/** Y Y | **___Kg**  **Missing** |  |
| D D **/** M M **/** Y Y | D D **/** M M **/** Y Y | **___Kg**  **Missing** |  |
| D D **/** M M **/** Y Y | D D **/** M M **/** Y Y | **___Kg**  **Missing** |  |
| D D **/** M M **/** Y Y | D D **/** M M **/** Y Y | **___Kg**  **Missing** |  |
| D D **/** M M **/** Y Y | D D **/** M M **/** Y Y | **___Kg**  **Missing** |  |
| D D **/** M M **/** Y Y | D D **/** M M **/** Y Y | **___Kg**  **Missing** |  |
| D D **/** M M **/** Y Y | D D **/** M M **/** Y Y | **___Kg**  **Missing** |  |
| D D **/** M M **/** Y Y | D D **/** M M **/** Y Y | **___Kg**  **Missing** |  |
| D D **/** M M **/** Y Y | D D **/** M M **/** Y Y | **___Kg**  **Missing** |  |
| D D **/** M M **/** Y Y | D D **/** M M **/** Y Y | **___Kg**  **Missing** |  |
| D D **/** M M **/** Y Y | D D **/** M M **/** Y Y | **___Kg**  **Missing** |  |
| D D **/** M M **/** Y Y | D D **/** M M **/** Y Y | **___Kg**  **Missing** |  |
| D D **/** M M **/** Y Y | D D **/** M M **/** Y Y | **___Kg**  **Missing** |  |
| D D **/** M M **/** Y Y | D D **/** M M **/** Y Y | **___Kg**  **Missing** |  |
| D D **/** M M **/** Y Y | D D **/** M M **/** Y Y | **___Kg**  **Missing** |  |
| D D **/** M M **/** Y Y | D D **/** M M **/** Y Y | **___Kg**  **Missing** |  |
| D D **/** M M **/** Y Y | D D **/** M M **/** Y Y | **___Kg**  **Missing** |  |
| D D **/** M M **/** Y Y | D D **/** M M **/** Y Y | **___Kg**  **Missing** |  |
| D D **/** M M **/** Y Y | D D **/** M M **/** Y Y | **___Kg**  **Missing** |  |
| D D **/** M M **/** Y Y | D D **/** M M **/** Y Y | **___Kg**  **Missing** |  |
| D D **/** M M **/** Y Y | D D **/** M M **/** Y Y | **___Kg**  **Missing** |  |
| D D **/** M M **/** Y Y | D D **/** M M **/** Y Y | **___Kg**  **Missing** |  |
| D D **/** M M **/** Y Y | D D **/** M M **/** Y Y | **___Kg**  **Missing** |  |
| D D **/** M M **/** Y Y | D D **/** M M **/** Y Y | **___Kg**  **Missing** |  |
| D D **/** M M **/** Y Y | D D **/** M M **/** Y Y | **___Kg**  **Missing** |  |
| D D **/** M M **/** Y Y | D D **/** M M **/** Y Y | **___Kg**  **Missing** |  |
| D D **/** M M **/** Y Y | D D **/** M M **/** Y Y | **___Kg**  **Missing** |  |
| D D **/** M M **/** Y Y | D D **/** M M **/** Y Y | **___Kg**  **Missing** |  |
| D D **/** M M **/** Y Y | D D **/** M M **/** Y Y | **___Kg**  **Missing** |  |
| D D **/** M M **/** Y Y | D D **/** M M **/** Y Y | **___Kg**  **Missing** |  |
| D D **/** M M **/** Y Y | D D **/** M M **/** Y Y | **___Kg**  **Missing** |  |
| D D **/** M M **/** Y Y | D D **/** M M **/** Y Y | **___Kg**  **Missing** |  |
| D D **/** M M **/** Y Y | D D **/** M M **/** Y Y | **___Kg**  **Missing** |  |
| D D **/** M M **/** Y Y | D D **/** M M **/** Y Y | **___Kg**  **Missing** |  |
| D D **/** M M **/** Y Y | D D **/** M M **/** Y Y | **___Kg**  **Missing** |  |
| D D **/** M M **/** Y Y | D D **/** M M **/** Y Y | **___Kg**  **Missing** |  |

If additional space is needed for **question 39,** please use the space below

| *Continuation from page 5, question 39* | | | | | |
| --- | --- | --- | --- | --- | --- |
| Date | Regimen | # days of prescription | Date | Regimen | # days of prescription |
| D D **/** M M **/** Y Y |  |  | D D **/** M M **/** Y Y |  |  |
| D D **/** M M **/** Y Y |  |  | D D **/** M M **/** Y Y |  |  |
| D D **/** M M **/** Y Y |  |  | D D **/** M M **/** Y Y |  |  |
| D D **/** M M **/** Y Y |  |  | D D **/** M M **/** Y Y |  |  |
| D D **/** M M **/** Y Y |  |  | D D **/** M M **/** Y Y |  |  |
| D D **/** M M **/** Y Y |  |  | D D **/** M M **/** Y Y |  |  |
| D D **/** M M **/** Y Y |  |  | D D **/** M M **/** Y Y |  |  |
| D D **/** M M **/** Y Y |  |  | D D **/** M M **/** Y Y |  |  |
| D D **/** M M **/** Y Y |  |  | D D **/** M M **/** Y Y |  |  |
| D D **/** M M **/** Y Y |  |  | D D **/** M M **/** Y Y |  |  |
| D D **/** M M **/** Y Y |  |  | D D **/** M M **/** Y Y |  |  |
| D D **/** M M **/** Y Y |  |  | D D **/** M M **/** Y Y |  |  |
| D D **/** M M **/** Y Y |  |  | D D **/** M M **/** Y Y |  |  |
| D D **/** M M **/** Y Y |  |  | D D **/** M M **/** Y Y |  |  |
| D D **/** M M **/** Y Y |  |  | D D **/** M M **/** Y Y |  |  |
| D D **/** M M **/** Y Y |  |  | D D **/** M M **/** Y Y |  |  |
| D D **/** M M **/** Y Y |  |  | D D **/** M M **/** Y Y |  |  |
| D D **/** M M **/** Y Y |  |  | D D **/** M M **/** Y Y |  |  |
| D D **/** M M **/** Y Y |  |  | D D **/** M M **/** Y Y |  |  |
| D D **/** M M **/** Y Y |  |  | D D **/** M M **/** Y Y |  |  |
| D D **/** M M **/** Y Y |  |  | D D **/** M M **/** Y Y |  |  |
| D D **/** M M **/** Y Y |  |  | D D **/** M M **/** Y Y |  |  |
| D D **/** M M **/** Y Y |  |  | D D **/** M M **/** Y Y |  |  |
| D D **/** M M **/** Y Y |  |  | D D **/** M M **/** Y Y |  |  |
| D D **/** M M **/** Y Y |  |  | D D **/** M M **/** Y Y |  |  |
| D D **/** M M **/** Y Y |  |  | D D **/** M M **/** Y Y |  |  |
| D D **/** M M **/** Y Y |  |  | D D **/** M M **/** Y Y |  |  |
| D D **/** M M **/** Y Y |  |  | D D **/** M M **/** Y Y |  |  |
| D D **/** M M **/** Y Y |  |  | D D **/** M M **/** Y Y |  |  |
| D D **/** M M **/** Y Y |  |  | D D **/** M M **/** Y Y |  |  |
| D D **/** M M **/** Y Y |  |  | D D **/** M M **/** Y Y |  |  |
| D D **/** M M **/** Y Y |  |  | D D **/** M M **/** Y Y |  |  |
| D D **/** M M **/** Y Y |  |  | D D **/** M M **/** Y Y |  |  |
| D D **/** M M **/** Y Y |  |  | D D **/** M M **/** Y Y |  |  |
| D D **/** M M **/** Y Y |  |  | D D **/** M M **/** Y Y |  |  |
| D D **/** M M **/** Y Y |  |  | D D **/** M M **/** Y Y |  |  |
| D D **/** M M **/** Y Y |  |  | D D **/** M M **/** Y Y |  |  |
| D D **/** M M **/** Y Y |  |  | D D **/** M M **/** Y Y |  |  |
| D D **/** M M **/** Y Y |  |  | D D **/** M M **/** Y Y |  |  |
| D D **/** M M **/** Y Y |  |  | D D **/** M M **/** Y Y |  |  |

If additional space is needed for **question 40,** please use the space below

| **Visit #** | **CD4** | **Date** | **VL** | **Date** | **Hgb** | **Date** | **ALT U/I** | **Date** | **Cr** | **Date** |
| --- | --- | --- | --- | --- | --- | --- | --- | --- | --- | --- |
| **13** |  | DD**/**MM**/**YY |  | DD**/**MM**/**YY |  | DD**/**MM**/**YY |  | DD**/**MM**/**YY |  | DD**/**MM**/**YY |
| **14** |  | DD**/**MM**/**YY |  | DD**/**MM**/**YY |  | DD**/**MM**/**YY |  | DD**/**MM**/**YY |  | DD**/**MM**/**YY |
| **15** |  | DD**/**MM**/**YY |  | DD**/**MM**/**YY |  | DD**/**MM**/**YY |  | DD**/**MM**/**YY |  | DD**/**MM**/**YY |
| **16** |  | DD**/**MM**/**YY |  | DD**/**MM**/**YY |  | DD**/**MM**/**YY |  | DD**/**MM**/**YY |  | DD**/**MM**/**YY |
| **17** |  | DD**/**MM**/**YY |  | DD**/**MM**/**YY |  | DD**/**MM**/**YY |  | DD**/**MM**/**YY |  | DD**/**MM**/**YY |
| **18** |  | DD**/**MM**/**YY |  | DD**/**MM**/**YY |  | DD**/**MM**/**YY |  | DD**/**MM**/**YY |  | DD**/**MM**/**YY |
| **19** |  | DD**/**MM**/**YY |  | DD**/**MM**/**YY |  | DD**/**MM**/**YY |  | DD**/**MM**/**YY |  | DD**/**MM**/**YY |
| **20** |  | DD**/**MM**/**YY |  | DD**/**MM**/**YY |  | DD**/**MM**/**YY |  | DD**/**MM**/**YY |  | DD**/**MM**/**YY |
| 21 |  | DD**/**MM**/**YY |  | DD**/**MM**/**YY |  | DD**/**MM**/**YY |  | DD**/**MM**/**YY |  | DD**/**MM**/**YY |
| 22 |  | DD**/**MM**/**YY |  | DD**/**MM**/**YY |  | DD**/**MM**/**YY |  | DD**/**MM**/**YY |  | DD**/**MM**/**YY |
| 23 |  | DD**/**MM**/**YY |  | DD**/**MM**/**YY |  | DD**/**MM**/**YY |  | DD**/**MM**/**YY |  | DD**/**MM**/**YY |
| 24 |  | DD**/**MM**/**YY |  | DD**/**MM**/**YY |  | DD**/**MM**/**YY |  | DD**/**MM**/**YY |  | DD**/**MM**/**YY |
| 25 |  | DD**/**MM**/**YY |  | DD**/**MM**/**YY |  | DD**/**MM**/**YY |  | DD**/**MM**/**YY |  | DD**/**MM**/**YY |
| 26 |  | DD**/**MM**/**YY |  | DD**/**MM**/**YY |  | DD**/**MM**/**YY |  | DD**/**MM**/**YY |  | DD**/**MM**/**YY |
| 27 |  | DD**/**MM**/**YY |  | DD**/**MM**/**YY |  | DD**/**MM**/**YY |  | DD**/**MM**/**YY |  | DD**/**MM**/**YY |
| 28 |  | DD**/**MM**/**YY |  | DD**/**MM**/**YY |  | DD**/**MM**/**YY |  | DD**/**MM**/**YY |  | DD**/**MM**/**YY |
| 29 |  | DD**/**MM**/**YY |  | DD**/**MM**/**YY |  | DD**/**MM**/**YY |  | DD**/**MM**/**YY |  | DD**/**MM**/**YY |
| 30 |  | DD**/**MM**/**YY |  | DD**/**MM**/**YY |  | DD**/**MM**/**YY |  | DD**/**MM**/**YY |  | DD**/**MM**/**YY |
| 31 |  | DD**/**MM**/**YY |  | DD**/**MM**/**YY |  | DD**/**MM**/**YY |  | DD**/**MM**/**YY |  | DD**/**MM**/**YY |
| 32 |  | DD**/**MM**/**YY |  | DD**/**MM**/**YY |  | DD**/**MM**/**YY |  | DD**/**MM**/**YY |  | DD**/**MM**/**YY |
| 33 |  | DD**/**MM**/**YY |  | DD**/**MM**/**YY |  | DD**/**MM**/**YY |  | DD**/**MM**/**YY |  | DD**/**MM**/**YY |
| 34 |  | DD**/**MM**/**YY |  | DD**/**MM**/**YY |  | DD**/**MM**/**YY |  | DD**/**MM**/**YY |  | DD**/**MM**/**YY |
| 35 |  | DD**/**MM**/**YY |  | DD**/**MM**/**YY |  | DD**/**MM**/**YY |  | DD**/**MM**/**YY |  | DD**/**MM**/**YY |
| 36 |  | DD**/**MM**/**YY |  | DD**/**MM**/**YY |  | DD**/**MM**/**YY |  | DD**/**MM**/**YY |  | DD**/**MM**/**YY |
| 37 |  | DD**/**MM**/**YY |  | DD**/**MM**/**YY |  | DD**/**MM**/**YY |  | DD**/**MM**/**YY |  | DD**/**MM**/**YY |
| 38 |  | DD**/**MM**/**YY |  | DD**/**MM**/**YY |  | DD**/**MM**/**YY |  | DD**/**MM**/**YY |  | DD**/**MM**/**YY |
| 39 |  | DD**/**MM**/**YY |  | DD**/**MM**/**YY |  | DD**/**MM**/**YY |  | DD**/**MM**/**YY |  | DD**/**MM**/**YY |
| 40 |  | DD**/**MM**/**YY |  | DD**/**MM**/**YY |  | DD**/**MM**/**YY |  | DD**/**MM**/**YY |  | DD**/**MM**/**YY |
| 41 |  | DD**/**MM**/**YY |  | DD**/**MM**/**YY |  | DD**/**MM**/**YY |  | DD**/**MM**/**YY |  | DD**/**MM**/**YY |
| 42 |  | DD**/**MM**/**YY |  | DD**/**MM**/**YY |  | DD**/**MM**/**YY |  | DD**/**MM**/**YY |  | DD**/**MM**/**YY |
| 43 |  | DD**/**MM**/**YY |  | DD**/**MM**/**YY |  | DD**/**MM**/**YY |  | DD**/**MM**/**YY |  | DD**/**MM**/**YY |
| 44 |  | DD**/**MM**/**YY |  | DD**/**MM**/**YY |  | DD**/**MM**/**YY |  | DD**/**MM**/**YY |  | DD**/**MM**/**YY |
| 45 |  | DD**/**MM**/**YY |  | DD**/**MM**/**YY |  | DD**/**MM**/**YY |  | DD**/**MM**/**YY |  | DD**/**MM**/**YY |
| 46 |  | DD**/**MM**/**YY |  | DD**/**MM**/**YY |  | DD**/**MM**/**YY |  | DD**/**MM**/**YY |  | DD**/**MM**/**YY |
| 47 |  | DD**/**MM**/**YY |  | DD**/**MM**/**YY |  | DD**/**MM**/**YY |  | DD**/**MM**/**YY |  | DD**/**MM**/**YY |
| 48 |  | DD**/**MM**/**YY |  | DD**/**MM**/**YY |  | DD**/**MM**/**YY |  | DD**/**MM**/**YY |  | DD**/**MM**/**YY |
| 49 |  | DD**/**MM**/**YY |  | DD**/**MM**/**YY |  | DD**/**MM**/**YY |  | DD**/**MM**/**YY |  | DD**/**MM**/**YY |
| 50 |  | DD**/**MM**/**YY |  | DD**/**MM**/**YY |  | DD**/**MM**/**YY |  | DD**/**MM**/**YY |  | DD**/**MM**/**YY |
| 51 |  | DD**/**MM**/**YY |  | DD**/**MM**/**YY |  | DD**/**MM**/**YY |  | DD**/**MM**/**YY |  | DD**/**MM**/**YY |
| 52 |  | DD**/**MM**/**YY |  | DD**/**MM**/**YY |  | DD**/**MM**/**YY |  | DD**/**MM**/**YY |  | DD**/**MM**/**YY |
| 53 |  | DD**/**MM**/**YY |  | DD**/**MM**/**YY |  | DD**/**MM**/**YY |  | DD**/**MM**/**YY |  | DD**/**MM**/**YY |
| 54 |  | DD**/**MM**/**YY |  | DD**/**MM**/**YY |  | DD**/**MM**/**YY |  | DD**/**MM**/**YY |  | DD**/**MM**/**YY |
| 55 |  | DD**/**MM**/**YY |  | DD**/**MM**/**YY |  | DD**/**MM**/**YY |  | | | DD**/**MM**/**YY |
